# Efficient identification of neoantigen-specific T-cell responses in advanced human ovarian cancer

**DOI:** 10.1186/s40425-019-0629-6

**Published:** 2019-06-20

**Authors:** Song Liu, Junko Matsuzaki, Lei Wei, Takemasa Tsuji, Sebastiano Battaglia, Qiang Hu, Eduardo Cortes, Laiping Wong, Li Yan, Mark Long, Anthony Miliotto, Nicholas W. Bateman, Shashikant B. Lele, Thinle Chodon, Richard C. Koya, Song Yao, Qianqian Zhu, Thomas P. Conrads, Jianmin Wang, George L. Maxwell, Amit A. Lugade, Kunle Odunsi

**Affiliations:** 1 Department of Biostatistics and Bioinformatics, Roswell Park Comprehensive Cancer Center, Buffalo, NY 14263 USA; 2 Center for Immunotherapy, Roswell Park Comprehensive Cancer Center, Buffalo, NY 14263 USA; 3 Gynecologic Cancer Center of Excellence, Department of Obstetrics and Gynecology, John P. Murtha Cancer Center, Uniformed Services University and Walter Reed National Military Medical Center, Bethesda, MD 20889 USA; 4 Department of Gynecologic Oncology, Roswell Park Comprehensive Cancer Center, Buffalo, NY 14263 USA; 5 Department of Cancer Prevention and Control, Roswell Park Comprehensive Cancer Center, Buffalo, NY 14263 USA; 6 Department of Obstetrics and Gynecology, Inova Fairfax Medical Campus, Falls Church, VA 22003 USA; 7 Inova Schar Cancer Institute, Falls Church, VA 22003 USA

**Keywords:** Neoantigen, Ovarian cancer, CD4^+^ T-cells, CD8^+^ T-cells, Anti-tumor effect, T-cell receptor, Gene therapy

## Abstract

**Background:**

Efficient identification of neoantigen-specific T-cell responses in epithelial ovarian cancer (EOC) remains a challenge. Existing investigations of spontaneous T-cell response to tumor neoepitope in EOC have taken the approach of comprehensive screening all neoantigen candidates, with a validation rate of 0.5–2%.

**Methods:**

Whole-exome and transcriptome sequencing analysis of treatment-naive EOC patients were performed to identify neoantigen candidates, and the immunogenicity of prioritized neoantigens was evaluated by analyzing spontaneous neoantigen-specfic CD4^+^ and CD8^+^ T-cell responses in the tumor and/or peripheral blood. The biological relevance of neoantigen-specific T-cell lines and clones were analyzed by evaluating the capacity of autologous ovarian tumor recognition. Genetic transfer of T-cell receptor (TCR) from these neoantigen-specific T-cell clones into peripheral blood T-cells was conducted to generate neoepitope-specific T-cells. The molecular signature associated with positive neoantigen T-cell responses was investigated, and the impacts of expression level and lymphocyte source on neoantigen identification were explored.

**Results:**

Using a small subset of prioritized neoantigen candidates, we were able to detect spontaneous CD4^+^ and/or CD8^+^ T-cell responses against neoepitopes from autologous lymphocytes in half of treatment-naïve EOC patients, with a significantly improved validation rate of 19%. Tumors from patients exhibiting neoantigen-specific T-cell responses exhibited a signature of upregulated antigen processing and presentation machinery, which was also associated with favorable patient survival in the TCGA ovarian cohort. T-cells specific against two mutated cancer-associated genes, *NUP214* and *JAK1*, recognized autologous tumors*.* Gene-engineering with TCR from these neoantigen-specific T-cell clones conferred neoantigen-reactivity to peripheral T-cells.

**Conclusions:**

Our study demonstrated the feasibility of efficiently identifying both CD4^+^ and CD8^+^ neoantigen-specific T-cells in EOC. Autologous lymphocytes genetically engineered with tumor antigen-specific TCR can be used to generate cells for use in the personalized adoptive T-cell transfer immunotherapy.

**Electronic supplementary material:**

The online version of this article (10.1186/s40425-019-0629-6) contains supplementary material, which is available to authorized users.

## Introduction

Epithelial ovarian cancer (EOC) is the deadliest gynecological cancer, with approximately 22,240 new cases and 14,070 deaths in the United States for 2018. Approximately 80% of EOCs are diagnosed at an advanced stage, for which the standard treatment is surgery followed by platinum-taxane chemotherapy. Despite the initial efficacy of these standard of care approaches, the overall five-year survival probability is only 28% [1], and there is an enormous unmet need for the development of alternative therapies. Association between improved clinical outcome and increased intraepithelial CD3^+^ and/or CD8^+^ tumor-infiltrating lymphocytes (TILs) suggested antitumor roles of T-cells in EOC [2–5]. High-affinity neoantigen-reactive T-cells can escape negative selection in the thymus and might have a greater potential to evoke a multi-pronged anti-tumor immune response due to the lack of central tolerance against them [6]. Indeed, recent correlative clinical studies indicate that T-cell reactivity to neoantigens is an important determinant of response to immune checkpoint inhibitors and other immunotherapies [7], suggesting that efforts to precisely define immunogenic neoantigens either for vaccination [8, 9] or adoptive T-cell therapy (ACT) [10, 11] could potentially provide clinical benefit [12].

A number of studies have reported T-cells specific to neoantigens in highly mutated tumors such as melanoma and lung cancer [8, 13–19]. Results from the existing studies of spontaneous T-cell response to tumor neoepitopes in EOC have been mixed [20–23]. Two earlier studies suggested that EOCs are rarely/unlikely to elicit neoepitope specific spontaneous T-cell response due to the relatively low somatic mutation burden [20, 21]. However, these studies were either limited by the small sample size (1–3 patients) or carried out in a murine ovarian tumor model, which has the concern of neoantigen being silenced and/or lost due to immune editing in the immunocompetent mouse. The studies were also restricted to CD8^+^ T-cells only, leaving the landscape of spontaneous CD4^+^ T-cell responses to tumor neoepitopes unexplored. Two more recent studies with larger sample size implied that by comprehensively screening all possible neoantigen candidates, identification of neoepitope specific T-cells is achievable in EOCs [22, 23]. Of the 1714 and 776 putative mutated neoantigens screened, less than 0.5 and 2% were shown to be immunogenic in validation experiments, respectively. Moreover, the capacity of autologous ovarian tumor recognition by neoantigen-specific T-cells has not been addressed.

The aim of this study was to investigate whether the validation rate of neoantigen identification in EOCs can be significantly improved through in silico prioritization. Whole-exome and transcriptome sequencing analysis of treatment-naive EOC patients were performed to identify neoantigen candidates, and the immunogenicity of prioritized neoantigens was evaluated by analyzing spontaneous neoantigen-specfic CD4^+^ and CD8^+^ T-cell responses in the tumor and/or peripheral blood. The biological relevance of neoantigen-specific T-cell lines and clones were analyzed by evaluating the capacity of autologous ovarian tumor recognition. Genetic transfer of T-cell receptor (TCR) from these neoantigen-specific T-cell clones into peripheral blood T-cells was conducted to generate neoepitope-specific T-cells. The molecular signature associated with positive neoantigen T-cell responses was investigated, and the impacts of expression level and lymphocyte source on neoantigen identification were discussed .

## Results

### Patient characteristics

The characteristics of the study cohort are shown in Additional file 12: Table S1. All 20 treatment-naïve patients underwent maximal debulking surgery (85% were optimally debulked, with 45% complete resection) from which the tumor tissue were procured. Patients in this cohort had typical characteristics of advanced EOC cases: median age at diagnosis of 60 (range 44 to 89), high stage (IIIC, IV; 100%), and the majority with high grade serous histology (75%). The median duration of follow-up was 29.7 months. The median progression-free survival was 18.1 months and the median overall survival was 30.9 months.

### Mutational landscape

**Fig. 1:**
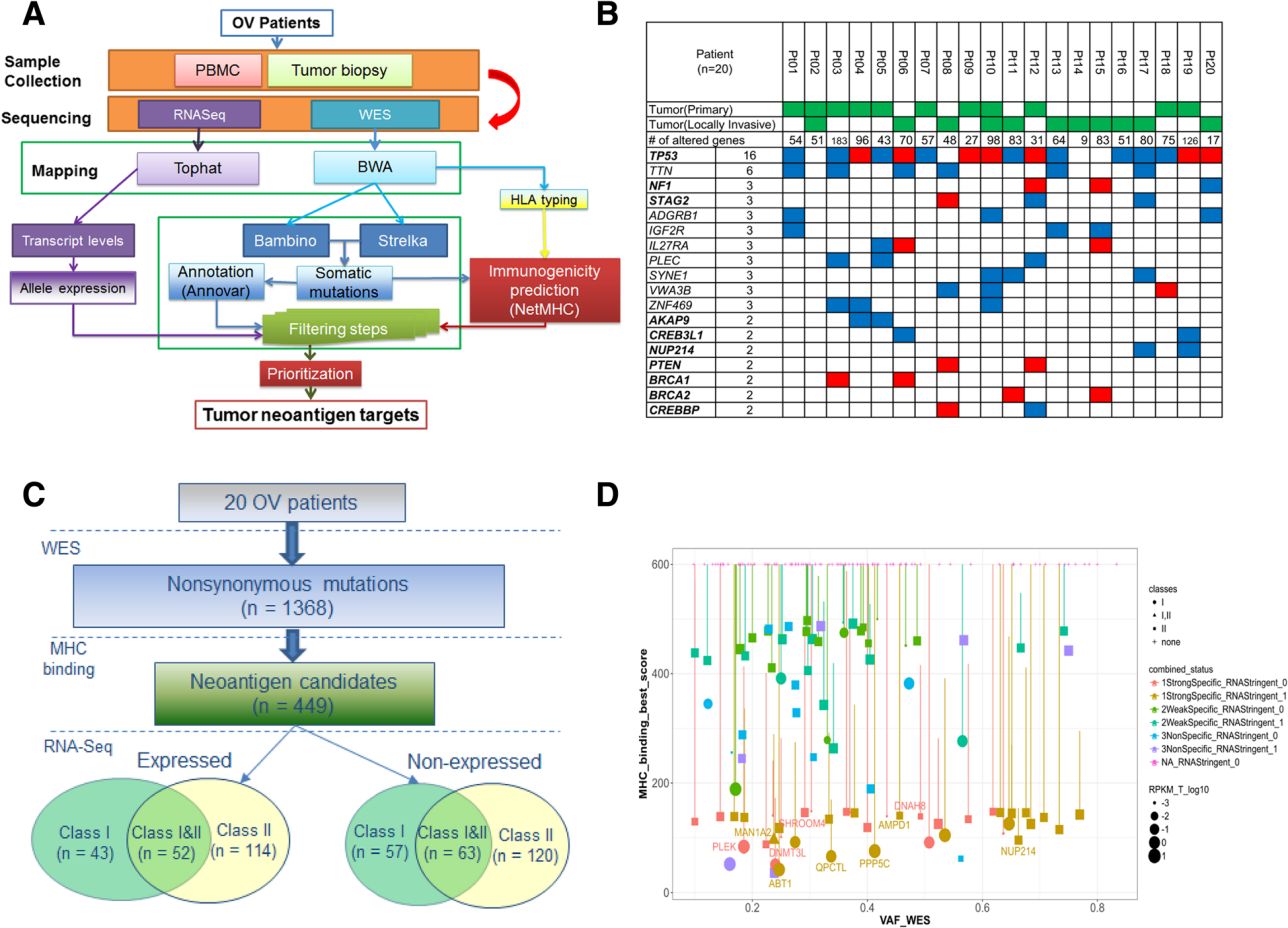
Integrative genomics and bioinformatics approach for neoantigen discovery and prioritization. **a** Overview of next-generation sequencing and neoantigen prediction. Whole-exome sequencing was performed on the pretreatment tumor and matched normal samples to identify somatic mutations, which were applied in neoantigen prediction pipeline that evaluates MHC binding, clonal status and gene expression to generate neoantigen specific to the patient’s HLA haploytype (Methods). **b** Top recurrently mutated genes in the 20 EOC patients, ordered by the numbers of recurrence. Known Cancer Gene Census (CGC) genes are in bold. For genes with recurrence at least 3, all genes are included. For genes with recurrence equals to 2, only known CGC genes are included. Red: truncating mutations, including nonsense SNV or frameshift Indels; Blue: altering mutations, including missense SNV or in-frame Indels. **c** Summary of neoantigen predictions in the 20 EOC patients, stratified by the MHC class type and gene expression status. There are 100 neoantigen predicted to bind to MHC class I only, 234 to class II only, and 115 to both class I and II, respectively. Among them, 209 are expressed based on RNAseq data. **d** The neoantigen landscape of Pt #19, as displayed in the Christmas Light Plot (CLP). The CLP incorporate pre-defined criteria for neoantigen prioritization, including MHC binding affinities, expression level, HLA class types, and the mutant clonal status. X-axis: Variant allele fraction (VAF) in WES, which can be used to infer clonal status; Y-axis: the predicted binding affinity of the mutant peptide. Each dot represents a neoantigen with the following characteristics displayed; size: the gene expression level by RNAseq; shape: HLA binding classes (I, II, or both); vertical bar: difference between mutant and wildtype binding affinities; color: stratified based on the mutant versus wildtype binding and mutant expression level (Methods). Gene symbols are displayed for neoantigens selected for screening

Whole-exome sequencing (WES) was performed on 22 pre-therapy biopsies and matched normal samples (Peripheral blood mononuclear cells, PBMC) from the 20 EOC patients, as the first step in our workflow for neoantigen discovery and prioritization (Fig. 1a). The specimens consisted of primary tumor in 9 patients, locally invasive tumor in 9 patients, paired primary and locally invasive tumors in 2 patients. Somatic mutations were identified by comparing the tumor with the matched blood DNA as described [24, 25]. We identified a total of 2096 somatic mutations from the 20 patients, including 1368 non-synonymous somatic mutations (median = 62), and the number of genes with altered amino acid sequence ranged from 9 to 183 per patient (Fig. 1b). *TP53* was mutated in 16 patients, including 7 truncating mutations predicted to cause loss-of-function (Additional file 1: Figure S1). Nine genes were mutated in 3 out of 20 patients, including two known Cancer Gene Census (CGC) genes [26]: *NF1* and *STAG2*. Of these nine genes, *IL27RA* appears to be interesting as two of the three mutations were truncating mutations. The two *IL27RA* loss-of-function mutations were both identified from locally invasive tumor while the third *IL27RA* missense mutation was found in primary tumor. In addition, there are 70 genes mutated in two patients, including seven CGC genes (Additional file 13: Table S2). *PTEN*, *BRCA1*, and *BRCA2* were each mutated in 2 patients, with all of them loss-of-function mutations. There was no gene found to be mutated at a significantly different frequency between primary and locally invasive tumors. In the two patients with both primary and locally invasive tumors, we compared every mutation’s variant allele fraction (VAF) between the two tumors and showed they were overall highly consistent (Additional file 2: Figure S2a).

### Identification of neoantigens

Candidate neoantigens were identified using the computational pipeline as outlined in Fig. 1a. We identified neoantigen candidates as mutations harboring mutant peptide whose binding affinity to patients’ MHC was not only strong (< 150 nM), but also specific (i.e., higher affinity than that of matched wild-type peptide). The second condition was included to enrich highly immunogenic neoepitopes because some neoepitope-reactive TCRs were considered to cross-react to the corresponding wild-type epitope if both mutated and wild-type epitopes were similarly presented [27]. In this case, T-cell precursors expressing such TCRs are expected to be eliminated in the thymus, which would result in lower precursor frequencies in the periphery. A total of 449 neoantigen candidates were found to have at least one predicted neopeptide with strong and specific MHC I and/or II-binding affinity. The number of predicted neoantigen in each patient ranged from 4 to 75, with a median of 21 (Additional file 14: Table S3). In the two patients with primary/locally-invasive tumor pair, the majority of neoantigens were shared by both primary and locally invasive tumors (100 and 77.8%, respectively) (Additional file 2: Figure S2b).

These 449 neoantigens included 215 which contained MHC I-binding neopeptides, and 349 which contained class II-binding neopeptides (Fig. 1c). One quarter (115 of 449) of neoantigens were predicted to harbor neopeptides which bind to both class I and II. We classified these 449 neoantigens based on the mutant allele’s expression level in RNAseq data (see Methods). About half (209) neoantigens demonstrated robust expression of the mutant allele while the rest (240) did not.

### Prioritization of neoantigens

To prioritize neoantigen candidates for peptide synthesis and T-cell assays, we ranked all predicted neoantigens within each patient based on a pre-defined set of criteria: 1) mutations in CGC genes; 2) MHC binding affinity of the mutant peptide; 3) difference in the binding affinities between mutant and the matched wild type peptides; 4) variant allele fraction (VAF) of the mutation; 5) expression level including both the mutant allele and the overall level of the gene; 6) type of MHC binding (class I only, class II only, or both class I and II). To facilitate this process, we designed a specific type of visualization plot (Christmas Light Plot, or CLP) incorporating all these types of information (Fig. 1d). The final selection of neopeptides involved a target selection board that evaluated the target peptides based on the criteria described above, with additional considerations of biochemical properties related to peptide synthesizability. For the ten patients with autologous PBMC, tumor-derived single-cell suspension and tumor biospecimens available, a total of 75 neopeptides were selected for synthesis, with a median of 7 and a range of 3–12 neopeptides per patient (Additional file 15: Table S4). These include 36, 32, and 7 neopeptides that are predicted to bind to class I only, class II only, and both class I and II, respectively. Twenty five of these 75 neopeptides did not demonstrate robust expression of the mutant allele in RNAseq (Methods), and they were included to investigate the relationship between expression level and induction of T-cell response.

### Evaluation of neoepitope immunogenicity

**Fig. 2:**
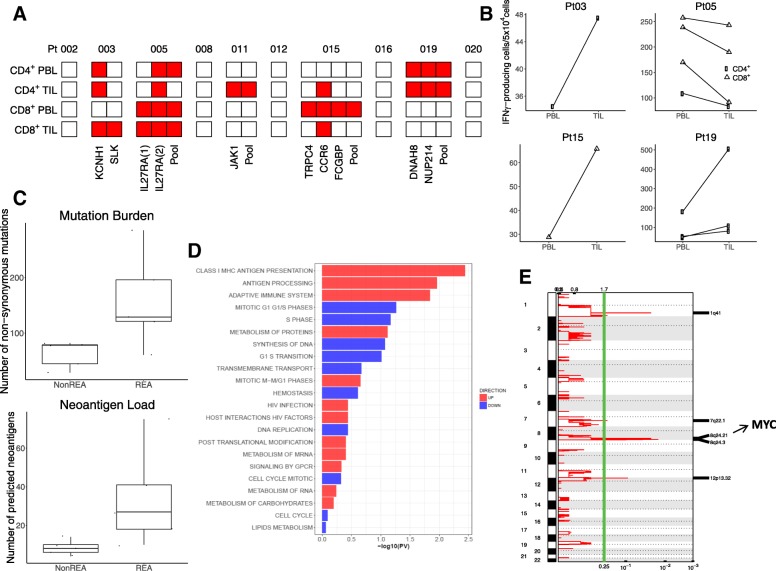
CD4^+^ and/or CD8^+^ T-cell response against neoepitopes in TILs and PBMCs. **a** The immunogenicity of the selected neoantigens was evaluated in the ten patients from whom both PBMCs and tumor biospecimens were available. Red and white squares indicate the presence and absence of spontaneous CD4^+^ and/or CD8^+^ T-cell response against mutant-specific epitopes, respectively. **b** In four patients, spontaneous CD4^+^ and/or CD8^+^ T-cell response against neoepitopes were detected in both TILs and PBMCs. T-cell reactivity was measured by IFN-γ ELISpot assay. **c** The mutation burden and neoantigen load of patients with mutant-specific T-cell response (RES) versus those without (NonRES). **d** The differentially enriched pathways between between patients with mutant-specific T-cell response and patients without. Up-regulated in red and down-regulated in blue. **e** Recurrent somatic copy number amplification in the patients without mutant-specific T-cell response. The genome is oriented vertically from top to bottom, and GISTIC q-values at each locus are plotted from left to right on a log scale. The green line represents the default significance threshold (q-value = 0.25)

The immunogenicity of the neoepitopes was evaluated in the ten patients from whom live T-cells from both PBMC and tumors were available. CD4^+^ and CD8^+^ T-cells were isolated from each specimen and stimulated with T-cell-depleted PBMCs as antigen-presenting cells (APCs) that had been pulsed with pooled neoepitopes. T-cell-depleted PBMCs were used to enrich APCs such as dentritic cells, monocytes/macrophages and B cells. A total of 27 IFN-γ-producing T-cell responses were detected in samples from 5 of 10 patients, including 20 responses against 10 individual neopeptide and 7 responses against 4 pooled neopeptides (Fig. 2a). These positive T-cell responses were highly mutant-specific, with the reactivity against mutated peptide at least two-fold greater than the corresponding wild-type peptide (Additional file 3: Figure S3 and Additional file 4: Figure S4). Both CD8^+^ and CD4^+^ T-cells showed neoepitope-specific responses, with 13 responses mediated by CD8^+^ T-cells and 14 by CD4^+^ T-cells. The median number of positive T-cell responses against individual neopeptides was 4 in the 5 responders, with a median of 2 reactive neoepitopes per patient.

For the 9 neopeptides (or neopeptide pools) that were recognized by CD8^+^ T-cells, 5 exclusively elicited mutant-specific T-cell response in either TILs or PBMCs. Likewise, 4 of the 9 neopeptides (or neopeptide pools) that were recognized by CD4^+^ T-cells exclusively elicited mutant-specific T-cell response in either TILs or PBMCs. This is consistent with prior evidence that there exists a discordance of neoepitope recognition between TILs and PBMCs [22]. Examples included the CD4^+^ T-cell response against mutant epitopes *of JAK1* which were detected only in TILs, and the CD8^+^ T-cell response against mutant epitopes *of TRPC4* which was present only in PBMCs (Fig. 2a). On the other hand, 18 of the 27 responses were detected in both TILs and PBMCs, indicating that both types of patient’s specimen are useful for identifying neoantigen-reactive T-cells (Fig. 2b). Examples include CD8^+^ and CD4^+^ T-cell responses against *IL27RA2* which were detetcted in both PBMC and TILs. Among the four patients with detected T-cell responses in both TILs and PBMCs, the responses were detected higher in TILs than PBMCs in three patients; while in Pt #5, there were more response in PBMCs than TILs (Fig. 2b). In order to explore immunosuppression status of TILs, we analyzed the expression level of a panel of 10 immune inhibitory molecules from the tumor RNA-Seq data of these four patients. These immune inhibitory molecules include *PD1*, *PDL1*, *CTLA4*, *CD80*, *CD86*, *LAG3*, *TIM3*, *LAGLS9*, *MYC* and *FOXP3*. Remarkably, Pt #5 showed higher expression of all these immune inhibitory genes than those of the other three patients (Additional file 5: Figure S5).

In total, 7/50 (14%) predicted neoepitopes that showed robust expression in RNAseq data induced T-cell responses. Interestingly, T-cell responses were also detected against 3/25 (12%) predicted neopeptides, *KCNH1* in Pt #3, *TRPC4* in Pt #15 and *DNAH8* in Pt #19, that were not robustly expressed in RNAseq (Additional file 15: Table S4). These 3 genes were all weakly expressed (with RPKM less than 1), although it is possible that the sequence depth of our RNAseq might not be sufficient to detect the mutant allele. It has been suggested that high level of expression may not be required for inclusion of a neoantigen candidate [28], based on the observations that very low levels (e.g., even a single peptide-HLA complex) may be sufficient for a cell to elicit a cytolytic T-cell response [29].

### Signatures of neoepitope-specific T-cell response

As expected, we found that patients with neoantigen-specific T-cell responses have significantly higher mutation burden and neoantigen load than those without (Fig. 2c). The median number of nonsynomous mutation is 84 in patients with responses, compared with 49 in patients without responses (*p* = 0.026, one-tailed t-test). The corresponding number of predicted neoantigen is 27 and 8, respectively (*p* = 0.043, one-tailed t-test). Interestingly, patients with positive T-cell responses are significantly enriched for *BRCA1/2* somatic mutations (3/5 vs 0/5, *p* = 0.038, chi-square test). When gene expression profiles for responders and non-responders were compared, the most significantly and differentially enriched pathways in responders were related to antigen processing and presentation machinery (APPM), suggesting that not only the number of neoantigens, but also antigen processing and presentation in tumor regulate generation of T-cell responses against neoantigens (Fig. 2d). On the other hand, the non-responders were characterized with a unique signature of *MYC* amplification (Fig. 2e**,** Additional file 6: Figure S6), which was recently shown to promote immune evasion through the modulation of immune regulatory molecules [30].

**Fig. 3:**
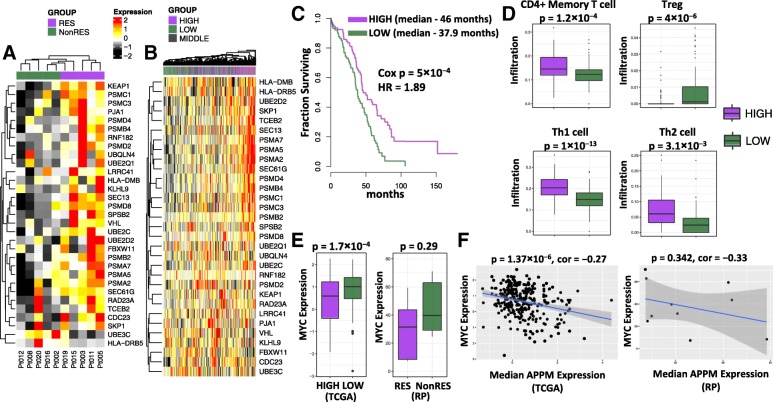
Molecular signatures of neoepitope-specfic T-cell response. **a**The signature of APPM, consisting of 31 APPM genes that are differentially expressed between patients with mutant-specific T-cell response (RES) and patients without (NonRES). **b** Stratificatioin of TCGA ovarian cancer patients into groups (tertiles) of high, middle and low expression of APPM signature, based on the median expression value of the signature in each patient. **c** Kaplan-Meier plot comparing TCGA ovarian cancer patients with high vs low expression level of APPM signature. **d** Comparision of tumor-infiltrating subtypes between TCGA ovarian cancer patients with high vs low expression level of APPM signature. **e** Expression of MYC in the TCGA cohort (in Z-score, left) comparing patients with high (purple) vs low (green) expression level of APPM signature, and in the Roswell Park (RP) cohort (in RPKM, right) comparing patients with (purple) vs without (green) mutant-specific T-cell response. **f** Correlation between the expression levels of APPM signature and *MYC* across the TCGA ovarian cancer patients (left) and Roswell Park (RP) patients (right)

By intersecting the list of differentially expressed genes (responders versus non-responders) with the genes of the APPM pathways, an 31-gene signature of APPM was derived (Fig. 3a**,** Additional file 16: Table S5). Based on median expression value of the APPM signature, we stratified TCGA ovarian cancer patients into groups of high vs low expression of this signature (Fig. 3b). In consistent with prior evidence that antigen presentation pathway is reduced in high-risk ovarian cancer [31], patients with higher expression of APPM signature have longer overall survival than patients with lower expression of the signature (*p* = 5 × 10^− 4^, Cox Proportional-Hazards Model) (Fig. 3c). Furthermore, patients with higher expression of APPM signature exhibited higher levels of CD4^+^ memory T-cells, Th1 and Th2 (*p* = 1.2 × 10^− 3^, 1 × 10^− 13^, and 3.1 × 10^− 3^, respectively, t-test), and lower level of Tregs (*p* = 4 × 10^− 6^, t-test) in tumors (Fig. 3d). On the other hand, patients with lower expression of APPM signature have a modest but significant increase in *MYC* expression (*p* = 1.7 × 10^− 4^, t-test) (Fig. 3e). Consistent with these findings, the TCGA ovarian cancer patients displayed a negative association between the expression levels of APPM signature and *MYC* (*p* = 1.37 × 10^− 6^, linear regression) (Fig. 3f). Similar trends were observed in the EOC patients from our study cohort, where the small sample size limited their statistical significance.

### Characterization of neoepitope-specific T-cells

In order to further characterize neoepitope-specific T-cells in EOC, we established neoantigen-specific T-cell lines by isolating and expanding peptide-reactive T-cells. T-cell lines that specifically recognized mutated peptides were established in 3 out of 9 cases we attempted. Based on the availability of autologous tumor specimens, we focused on *NUP214* neoepitope-specific CD4^+^ T-cells obtained from TILs of Pt #19 and *JAK1* neoepitope-specific CD4^+^ T-cells obtained from TILs of Pt #11.

**Fig. 4:**
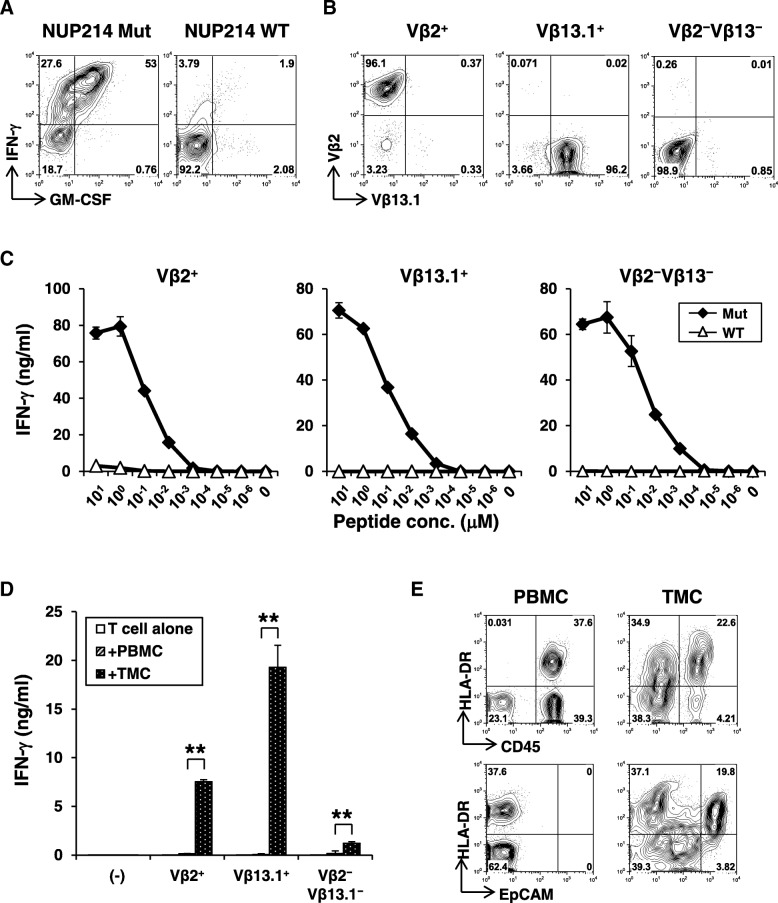
Characterization of *NUP214* neoepitope-specific CD4^+^ T-cells. **a** Peptide reactivity of a *NUP214* neoepitope-specific CD4^+^ T-cell line. IFN-γ and GM-CSF production on CD4^+^ T-cells against mutated or wild-type NUP214 peptide-pulsed autologous EBV-transformed B (EBV-B) cells were determined by intracellular cytokine staining. **b** Establishment of NUP214 neoepitope-specific CD4^+^ T-cell clones. TCR Vβ2^+^, Vβ13.1^+^, or Vβ2^−^Vβ13.1^−^ cells in the NUP214 neoepitope-specific CD4^+^ T-cell lines were isolated. After expansion, each T-cell clone was stained by TCR Vβ subtype-specific antibodies. **c** Avidity of NUP214 neoepitope-specific T-cell clones. Vβ2^+^, Vβ13.1^+^, and Vβ2^−^Vβ13.1^−^ CD4^+^ T-cell clones (50,000 cells) were stimulated with autologous EBV-B cells (25,000 cells) pulsed with NUP214 mutated or wild-type peptide in a 96-well round bottom plate for 24 h. IFN-γ level in the culture supernatant was measured by ELISA. The data represents mean ± s.d. of duplicate wells. **d** Reactivity of Vβ2^+^, Vβ13.1^+^, and Vβ2^−^Vβ13.1^−^ T-cell clones against autologous tumor cells. PBMCs or TMCs (100,000 cells) were co-cultured with Vβ2^+^, Vβ13.1^+^, or Vβ2^−^Vβ13.1^−^ NUP214 neoepitope-specific CD4^+^ T-cells (50,000 cells) or without T-cells (−) for 24 h. TMCs: tumor tissue-derived mononuclear cells. IFN-γ production was measured by ELISA. The data represent mean + s.d. of triplicate wells. **p < 0.01 (student’s t-test) compared to IFN-γ level against PBMCs. **e** Expression of MHC class II on CD45^+^ immune cells and EpCAM^+^ tumor cells. HLA-DR expression on CD45^+^ or EpCAM^+^ cells from PBMCs or TMCs were analyzed by flow cytometry

About 80% of neoepitope-specific CD4^+^ T-cell line produced IFN-γ against the mutated NUP214 peptide but not the corresponding wild-type peptide (Fig. 4a). Low-resolution TCR Vβ spectratyping identified the CD4^+^ T-cell response as oligoclonal (Additional file 7: Fig. S7a), composed of 20% Vβ2^+^ and 45% Vβ13.1^+^ T-cells, respectively. Approximately 30% of cells were of other Vβ subtype not identified by this antibody panel. Combination of Vβ-staining and intracellular IFN-γ staining demonstrated that all 3 distinct major subsets recognized the same neopeptide (Additional file 7: Fig. S7b). Therefore, TCR Vβ2^+^, Vβ13.1^+^, or Vβ2^−^Vβ13.1^−^ cells were further isolated by flow cytometric cell sorting to obtain clonal populations (Fig. 4b). Avidity for recognition of mutated peptide by Vβ2^+^, Vβ13.1^+^, and Vβ2^−^Vβ13.1^−^ *NUP214*-specific CD4^+^ T-cell clones was similar (Fig. 4c). Responses were strictly specific for mutation as there was no recognition of wild-type peptide even at higher concentrations. As *NUP214*-specific CD4^+^ T-cell responses was detected in the tumor from Pt #19 (Fig. 2 a), we reasoned that the mutated *NUP214* epitope was naturally presented in the tumor microenvironment. Therefore, we tested whether *NUP214*-specific CD4^+^ T-cells are activated by autologous tumor cells. Indeed, *NUP214* neoepitope-specific CD4^+^ T-cells produced IFN-γ specifically against tumor cells, but not against autologous PBMCs (Fig. 4d). These results strongly support that *NUP214*-specific CD4^+^ T-cells were activated in the tumor microenvironment. In Pt #19 tumor, both CD45^+^ hematopoietic cells and EpCAM^+^ EOC expressed MHC class II (HLA-DR) (Fig. 4e). Therefore, both direct presentation by cancer cells and indirect cross-presentation of tumor-derived *NUP214* by hematopoietic antigen-presenting cells are possible as a mechanism for activation of mutated *NUP214*-specific CD4^+^ T-cells in the tumor microenvironment.

The *JAK1* neoepitope-specific CD4^+^ T-cells were isolated and expanded (Additional file 8: Figure S8a) and were analyzed for TCR Vβ usage (Additional file 8: Figure S8b). The majority (75%) of JAK1-specific CD4^+^ T-cell line was Vβ13.6^+^, indicating a monoclonal response. TCR Vβ13.6^+^ CD4^+^ T-cells were further isolated and expanded for functional analyses (Additional file 8: Fig. S8c). Vβ13.6^+^ CD4^+^ T-cells specifically recognized JAK1 mutant peptide over the corresponding wild-type peptide (Additional file 8: Figure S8d). Because autologous tumor mononuclear cells (TMCs) were not available for this patient, we tested the reactivity against tumor ascites mononuclear cells (AMCs) and found that CD4^+^ T-cells produced IFN-γ when co-cultured with the AMCs but not with the autologous PBMCs (Additional file 8: Figure S8e).

### Generation of neoepitope-specific T-cells by TCR gene-engineering

**Fig. 5:**
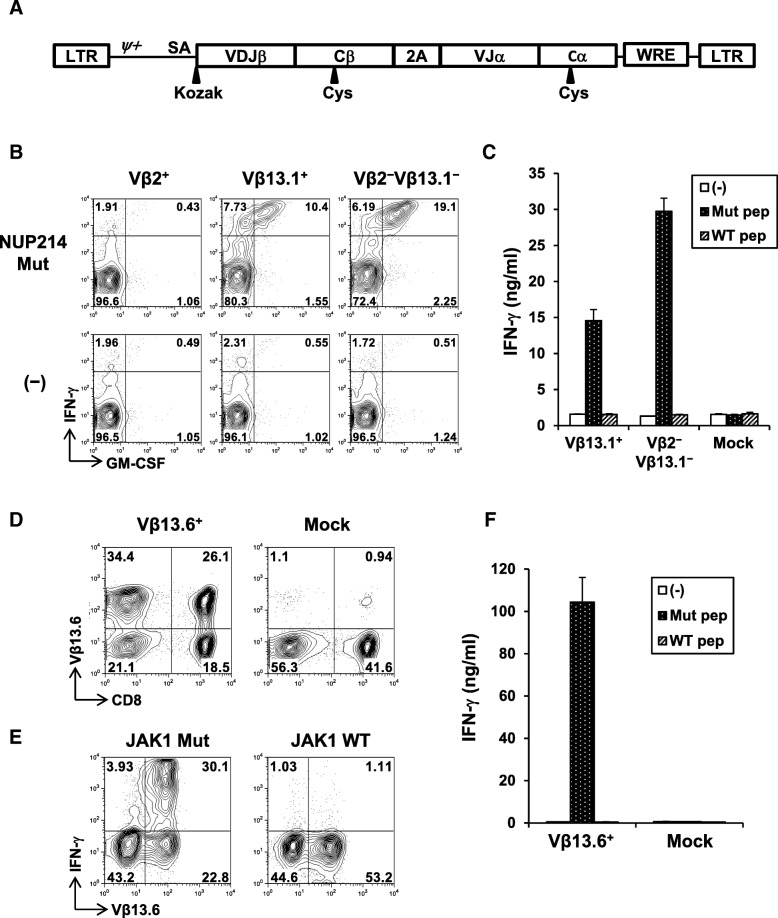
Generation of neoantigen-specific CD4^+^ T-cells by TCR gene-engineering. **a** Schematic representation of retroviral TCR expression vector for TCR gene-engineering. LTR: long terminal repeats; *ψ*^*+*^: extended packaging signal; *SA*: Splice acceptor site from the first exon-intron junction of human elongation factor-1α; Kozak: Kozak consensus sequence (GCCACC); VDJβ: TCR β chain variable-diverse-joining regions; Cβ: TCR β chain constant region containing a Cystein modification; 2A: the P2A translational skipping sequence; VJα: TCR α chain variable-joining regions; Cα: TCR α chain constant region containing a Cystein modification; and WRE indicates the: Woodchuck hepatitis virus posttranscriptional regulatory element. **b-c** T-cell function of NUP214-specific TCR-transduced T-cells. **b** IFN-γ and GM-CSF production from Vβ2^+^, Vβ13.1^+^, or Vβ2^−^Vβ13.1^−^ TCR-transduced T-cells against autologous EBV-B cells pulsed with or without NUP214 mutated peptide. **c** IFN-γ production from Vβ13.1^+^ or Vβ2^−^Vβ13.1^−^ TCR transduced T-cells against NUP214 mutated or wild-type peptide was measured by ELISA. Mock: TCR-untransduced T-cells. **d-f** Transduction efficiency and function of JAK1 neoepitope-specific TCR-transduced T-cells. **d** Vβ13.6^+^ TCR transduction efficiency was examined by flow cytometry. **e** Detection of JAK1 neoepitope-specific response on Vβ13.6^+^ T-cells by intracellular cytokine staining. **f** Reactivity of TCR-transduced T-cells against JAK1 mutated or wild-type peptide was tested by ELISA

To test whether neoantigen-reactivity is solely mediated by TCR and whether neoantigen-specificity can be transferred to other T-cells by TCR gene-engineering, we first cloned TCR gene from 5 neoepitope-specific T-cell clones (3 mutated NUP214-specific CD4-TCR from Pt #19, 1 JAK1-specific CD4-TCR from Pt #11, and 1 TRPC4-specific CD8-TCR from Pt #15) into a retroviral plasmid vector (Fig. 5a) [32]. To test the functionality of the cloned TCR, peripheral T-cells from a healthy donor were polyclonally activated and transduced by the TCR-expressing retroviral vectors. In 4/5 cases, TCR gene-engineered T-cells were demonstrated for mutated peptide-specific reactivity. In the case of 3 *NUP214*-specific TCRs, two TCRs from Vβ13.1^+^ or Vβ2^−^Vβ13.1^−^ T-cell clones provided neoepitope-specific reactivity (Fig. 5b-c), while that from Vβ2^+^ T-cell clone did not despite of similar reactivity by the parental T-cell clones (Fig. 4c). Both CD4^+^ and CD8^+^ T-cells transduced with NUP214-specific TCR showed reactivity against the neoepitope (Additional file 9: Figure S9). Similar observations were made for mutated JAK1-specific TCR, where we have established high-titer retrovirus-packaging PG13 cell clone. After 2 transductions, nearly 60% T-cells expressed transduced TCR, as determined by the increase in TCR Vβ13.6^+^ expression (Fig. 5d). Mutated JAK1-specific TCR-transduced T-cells also displayed strong and specific reactivity against the mutated JAK1 peptide (Fig. 5e-f). In addition to CD4^+^ T-cells, we also cloned TCR gene from *TRPC4* neoepitope-specific CD8^+^ T-cells, and confirmed the neoantigen reactivity by TCR gene-engineered T-cells (Additional file 10: Figure S10).

## Discussion

Advanced EOC is a highly lethal disease, with a dismal five-year survival rate below 30% [1]. The strong association of TILs with better survival [33], which is counter-regulated by innate and adaptive immune escape mechanisms such as expression of immunosuppressive molecules and recruitment of immunosuppressive cells, indicates that patients with advanced EOC might benefit from immunotherapy. Emerging immunotherapies targeting patient-specific neoantigens have attracted considerable interest because immune responses to tumor-specific neoantigens are less constrained by central and peripheral immune tolerance mechanisms [34].

Given the relatively lower mutation burden of EOC than melanoma and lung cancer, previous investigations of spontaneous T-cell response to tumor neoepitope in EOC have taken the approach of comprehensively screening all identified neoantigen candidates [20–23]. For example, Bobisse et al screened all 776 predicted HLA class I-binding neopeptide individuals as well as 19 neopeptide pools from 19 patients (mean 46 per patient) and validated 15 of them, or 2% [22]. Dinger et al screened all 1714 putative mutated neoantigens from 7 patients (mean 214 per patient) and validated 8 of them, or 0.5% [23]. In contrast, we only screened a subset (mean 9 per patient) of neopeptide candidates prioritized by our in silico approach, and achieved a substantially higher validation rate (14 of the 75 neopeptide individuals and 10 neopeptide pools, or 19%) for practical use, especially when limited patient-derived specimens are available. On the other hand, our approach might have missed some bona fide neoantigen targets, and therefore underestimated the prevalence of neoepitope specific T-cells in patients with EOC.

As expected, patients exhibiting T-cell responses against neoepitopes were found to have significantly higher mutation burden and neoantigen load. In addition, these responders tended to have higher BRCA1/2 mutation rate than those without. Due to the relatively low mutation burden of EOC and widespread interpatient tumour heterogeneity, our prioritization approach will more likely be applicable to a subset of patients of EOC. In addition, since somatic mutations can vary at different times in tumor evolution (e.g., primary tumor versus tumor relapse), it will be desirable to repeat this procudure sequentially to identify new neoepitope-specific T cells.

We found elevated level of APPM signature in patients who spontaneously developed neoepitope-specific T-cell response, while MYC amplification was observed in patients who did not. Among the five responders in our study, Pt #5 has lower mutation burden than three of the five non-responders (45 vs 49–52, Fig. 2c). Interestingly, the expression level of APPM signature is almost two-fold higher in Pt #5 than the three non-responders (Additional file 11: Figure S11). Our results provided independent edvidence that antigen presentation pathway is associated with ovarian cancer prognosis [31], and lends additional support for the crucial role of this machinery in TIL infiltration and defining CD8^+^/CD4^+^ T-cell responses against neoantigens.

The biological relevance of patient-derived T-cells against neoantigens from known cancer-associated genes, *NUP214* and *JAK1,* were confirmed by the demonstration of autologous tumor recognitions*. NUP214* encodes a protein localized to the cytoplasmic face of the nuclear pore complex where it is required for proper cell cycle progression and nucleocytoplasmic transport. *NUP214* forms a fusion gene with the *DEK* gene on chromosome 6 in a t [6, 9] translocation associated with acute myeloid leukemia and myelodysplastic syndrome [35, 36]. Somatic mutations in *JAK1/2* have been proposed as a mechanism of evasion to immune recognition, leading to both primary and acquired resistance to *PD-1* blockade in some cancer patients [37, 38]. Pre-clinical studies have suggested that targeting *JAK/STAT3* could effectively suppress ovarian tumor progression and have therapeutic potential for treating advanced EOC [39–41]. Our observations suggest that although cancer cells may evade immunological attack by mutating genes such as *JAK1*, de novo T-cell responses against such mutations might be exploited to inhibit tumor growth in cancer patients. Interestingly, we discovered *IL27RA* mutations in 3 patients, including two loss-of-function truncating mutations and one missense mutation. The *IL27RA* missense mutation was shown to elicit concomitant CD4^+^ and CD8^+^ T-cell responses. *IL27RA* is the alpha subunit of Interleukin 27 (*IL27*) receptor, which has been reported to demonstrate a dual role of anti-tumor activity and immune regulatory function [42, 43]. A recent proteomic analysis on human ovarian cancer cell lines revealed that *IL-27* and *IFN-γ* shared a broad set of activities, such as the HLA class I antigen presentation [44]. Loss of the IFN-γ pathway gene has been shown as a mechanism of resistance to anti-CTLA-4 therapy [45]. It is possible that mutations in *IL27RA* provide a novel immune-evasion mechanisms in EOC. Further validations and functional studies will be needed to determine the prognostic and therapeutic potential of *IL27-IL27R* pathway genes in EOC.

Our study reveals that a significant portion of neoantigen candidates can fail to pass the predefined criteria for mutant allele expression in RNAseq, and we showed some of them could elicit strong mutant-specific CD4^+^ and/or CD8^+^ T-cell response in autologous PBMCs and/or TILs. Conceptually, the level of expression of the source protein is a surrogate for determining whether it reaches the threshold required for its efficient processing and presentation in HLA, and whether neoantigen-specific T-cells would efficiently reciognize the tumor target. The latter is mainly determined by affinity of TCR to interact with MHC/neoepitope complex. For virus antigen-specific T-cells, only a single MHC/peptide is sufficient to activate T-cells through high-affinity TCRs [46]. Because affinity for neoantigen-specific TCRs is considered to be high due to absence of central tolerance, it is possible that low level of expression is enough to induce neoantigen-specific T-cell responses. There have been multiple strategies to use expression information for neoantigen prediction, including studies using RNAseq from autologous tumor samples [8], studies using RNAseq data from different patients of the same cancer type (i.e., TCGA) [47], and studies not using expression information [22, 28, 48]. Even for the strategy of using RNAseq from autologous tumor samples, the criteria used by different studies to determine neoantigen expression can be vastly different. Therefore, it will be necessary in the future to conduct comprehensive assessments of the role of expression information and the approaches of leveraging it for neoantigen prediction and/or prioritization.

The nature of relatively low mutation burden of EOC also calls for culture enrichment approaches to expand neoepitope specific clones. In the study by Bobisse et al [22], whole tumors were cultured with high concentration of IL-2 in the presence MHC class I neoepitope pools [22]. In our study, we stimulated isolated CD4^+^ and CD8^+^ T-cells from tumors with peptide-pulsed CD4^−^CD8^−^ cells derived from PBMCs, because APCs in ovarian tumor microenvironment have been shown to be dysfunctional or immunosuppressive [49]. A recent study by Yossef et al showed that the detection of neoantigen-reactive TILs could be enhanced by enriching T-cells that express PD-1 and/or T-cell activation markers followed by microwell culturing to avoid overgrowth of nonreactive T-cells [50]. As the availability of patients’ specimens such as TILs is often limited, future studies are warranted to benchmark the prediction accuracy and effectiveness of different culture enrichment approaches for screening of immunogenic neoantigens in EOC.

We were able to isolate and clone TCRs from in vitro expanded CD4^+^ and CD8^+^ T-cell clones reactive against neoepitopes, and demonstrated the feasibility of specifically targeting the neoantigens by TCR gene-engineered T-cells. Our current protocoal takes about 2 weeks to prepare TCR-transduced T-cells from the established neoantigen-specific T-cell lines. Adoptive transfer of autologous tumor antigen-specific T-cells is an effective therapeutic treatment for cancer patients [51, 52]. Using tumor antigen-specific TCR genes, tumor antigen-specific T-cells can be rapidly expanded for infusion into patients in order to mediate immediate elimination of tumors and long-term tumor immune-surveillance. The therapeutic effects of ACT using TCR gene-engineered T-cells have been demonstrated in many clinical trials. High-affinity TCR gene is required to engineer effective T-cells for strong recognition and destruction of cancer cells. It has been recently reported that function of neoepitope-specific CD8^+^ T-cell from ovarian tumors is significantly higher than PBMCs [22], suggesting neoepitope-specific CD4^+^ and/or CD8^+^ T-cells in TILs could be a useful source of TCR for ACT.

The neoepitope-specific TCRs could be introduced into naïve or central memory T-cells to target driver mutations [11], simultaneously target multiple mutations, and combine ACT with other immunomodulators such as checkpoint inhibitors. Conceptually multiple neoantigens can be targeted but it is difficult to define an optimal number of neoepitopes. The range of neoepitopes utilized in published clinical trials of neoantigen vaccines varies between 7 and 20 [8, 9, 53]. Due to the intratumoral heterogeneity, it would be desirable to target multiple neoantigens for each patient. Although most previous ACT trials using TCR gene-engineered T cells targeted a single tumor antigen epitope, it is feasible to target multiple tumor antigens using a mixture of engineered T-cell products. As assessment of the immunosuppressive microenvironment was not the main focus of our current study, we did not include exogenous checkpoint blockade (e.g. anti-CTLA4 or anti-PD1) into our neoantigen recognition assays. It is well accepted that immunosuppression within the tumor microenvironment represents a critical barrier for anti-tumor T cell function, both neoantigen-reactive and adoptively transferred engineered T cells. A published report from a recent clinical trial evaluating adoptive transfer of TCR-engineered T cells and ipilimumab (anti-CTLA4) demonstrated that while addition of ipilimumab was safe and tolerable, there was no apparent clinical benefit from the combination [54]. Although the small sample size (*n* = 4) was insufficiently powered, this study and other preclinical/clinical studies suggest immunomodulatory combinations (e.g. anti-PD1, anti-TGF-β, IDO inhibitors) could still potentiate therapeutic effect of ACT. Additional studies are required to empirically determine the optimal combination for eliciting clinical benefit. Our current study focused on analyzing treatment-naïve ovarian cancer patients at the time of primary debulking surgery. Future studies will be warranted to determine intrinsic factors of tumor and microenvironment as well as the nature of TCR affecting the infiltration of adoptively transferred T cells. In future ACT clinical trials using TCR gene-engineered T cells, it is also feasible to quantify engineered T cells in the tumors using quantitative TCR or digital PCR. Taken together, these results warranted future studies of patient-specific neoantigens as potential targets for downstream translational validation towards adoptive T-cell immunotherapy of ovarian cancer.

## Conclusion

In summary, our study demonstrated the feasibility of efficiently identifying both CD4^+^ and CD8^+^ neoantigen-specific T-cells in ovarian cancer. Further methodology development [55], including the improvement of patient response prediction, neoantigen prediction and prioritization, T-cell enrichment and TCR engineering, will be warranted to exploit the therapeutic potential of neoantigen-targeting for personalized immunotherapy in treating advanced EOC.

## Methods

### Patient and sample characteristics

Tumor specimens were collected at the time of primary debulking surgery, flash frozen in liquid nitrogen and stored at − 80 °C. Portions of tumor specimens were dissociated by the Gentle MACS system (Miltenyi Biotec) to obtain single-cell suspensions. After a density gradient isolation, live mononuclear cells were stored in liquid nitrogen until use. PBMC were obtained using a density gradient method and stored in liquid nitrogen. DNA was extracted from the frozen tissues and PBMCs using the GenFIND DNA extraction kit (Agencourt, Pasadena, CA) per the manufacturer’s instructions. All pathology specimens were reviewed by experienced gynecologic pathologists and tumors were classified according to the WHO criteria [56]. Prior to surgery, no patients received neo-adjuvant chemotherapy, and subsequent to surgery all patients received adjuvant platinum/taxane-based chemotherapy. No patient had received immunotherapy treatment prior to surgery.

### Whole-exome sequencing and somatic mutation calling

Individual exome capture of each DNA sample followed by single-indexed library generation was carried out using the SureSelect XT Target Enrichment System (Human All Exon V5 kit, Agilent Inc). Cluster generation on cBot was followed by 2 × 100 bp paired-end (PE) sequencing on a HiSeq2500 sequencer according to the manufacturer’s recommended protocol (Illumina Inc.). High quality WES paired-end reads passing Illumina RTA filter were aligned to the NCBI human reference genome (GRCh37) using Burrows-Wheeler Alignment (BWA) tool [57]. PCR duplicated reads were marked and removed using Picard tool. All samples had more than 80% of the targeted regions covered by at least 30-fold. Putative mutations were identified by running variation detection module of Bambino [58] and Strelka [59], and then further filtered as previously described [25, 60]. All putative single-nucleotide variants (SNVs) were combined and further filtered based on a standard set of criteria to remove the following common types of false calls: [1] the alternative allele is present in the matching normal sample and the contingency between the tumor and normal samples is not statistically significant; [2] the mutant alleles are only present in one strand and the strand bias is statistically significant; [3] the putative mutation occurs at a site with systematically dropped base quality scores; [4] the reads harboring the mutant allele are associated with poor mapping quality. The identified somatic mutations were compared to the public human germline databases including dbSNP [61], 1000 Genomes Project [62], NHLBI’s Exome Sequencing Project to further exclude remaining germline polymorphisms. All mutations were manually reviewed to ensure accuracy and annotated using ANNOVAR [63] with the latest NCBI RefSeq database. To extract somatic copy number information based on the sequenced exomes of the samples, Varscan2 [64] was employed, and the results were smoothed and segmented with the Bioconductor DNACopy package [65]. GISTIC2.0 [66] was used to identify recurrent somatic chromosomal alterations in the five samples with mutant-specific T-cell response and the five samples without, respectively.

### RNAseq and gene expression analysis

RNAs were purified from fresh frozen tumors using the miRNeasy mini kit (Qiagen). The sequencing libraries were prepared with the TruSeq Stranded Total RNA kit (Illumina Inc) and sequenced for 100 cycle paired-end on a HiSeq2500 sequencer, following the manufacturer’s recommended protocol (Illumina Inc.). Raw reads from RNAseq that passed the Illumina RTA quality filter were first pre-processed using Cutadapt to remove adapter sequences, followed by FASTQC for sequencing base quality control. The remaining reads were mapped to the NCBI human reference genome (GRCh37) and RefSeq annotation database using Tophat [67]. A second round of quality control was performed to identify potential RNAseq library preparation problems by examining mapped BAM files using RSeQC [68]. From the mapping results, the number of reads aligning to each gene was calculated using HTSeq [69] and RPKM (Reads Per Kilobase of transcript per Million mapped reads) values were obtained using RSEM [70]. ssGSEA [71], an extension of Gene Set Enrichment Analysis [72], was performed on gene expression data of each sample using the Reactome gene sets obtained from the MSigDB database [73]. The normalized enrichment score of each gene set in each sample was retrieved, and compared between the five samples with mutant-specific T-cell response and the five samples without. Differentially expressed genes (DEGs) between those patients who responded and those who didn’t were determined using LIMMA [74]. By intersecting the list of DEGs (*p* < 0.05) with the genes involved in the antigen processing and presentation machinery (APPM) pathways, a signature of APPM constsiting of 31 DEGs were obtained. TCGA gene expression dataset were downloaded from cBioportlal [75] using Ovarian Serous Cystadenocarcinoma (TCGA Provisional, 307 samples with RNAseq). TCGA ovarian cancer patients were ranked based on median expression value of the 31-gene signature and grouped in top vs bottom tertile (groups of high versus low expression level of signature). Survival analysis was done using the survival package in R. Precalculated dataset of tumor-infiltrating subtypes for the TCGA Ovarian cancer patients were downloaded from xCell [76] and the comparsion of tumor-infiltrating subtypes between groups of high versus low expression level of signature was performed using Student’s t-test. Correlation between the expression of APPM signature and *MYC* was calculated using Pearson correlation, and a linear model was built to fit the data and test significance and was plotted as trendline with the confidence intervals.

### Neoantigen prediction

For each missense SNVs, we obtained 8 to 15-mer peptide sequences containing the mutated amino acid as well as corresponding wild-type ones from RefSeq [77]. Genotypes for patients’ class I and II HLA alleles were determined from next-generation sequencing data using Polysolver and HLAminer, respectively, with default parameters [78, 79]. Class I and II-binding affinities for each combination of peptide/HLA type were predicted using NetMHCpan v3.0 [80–82] and NetMHCIIpan v3.1 [80], respectively, with default parameters. For a mutant peptide to be considered as a neoepitope, we required: 1) mutant binding affinity score is less than 150 nM; 2) the ratio of binding affinity between mutant and the matched wild-type peptides is less than 0.9; and 3) the difference in binding affinity between mutant and wild-type epitopes is at least 100 nM, except for peptides from any Cancer Census genes [26]. If one mutation was predicted to generate multiple neoepitopes, it was counted as one neoantigen [48]. For the predicted neoantigen, we assessed the expression of mutant allele in RNAseq using a set of previously published criteria for neoantigen expression [83]: 1) at least two supporting reads in RNAseq; 2) minimum variant allele fraction (VAF) of 4% for mutations with at least three reads or 20% for mutations with exact two supporting reads; 3) no significant strand bias (*p* < 0.05).

### Peptide synthesis

Synthetic peptides for neoepitopes and the corresponding wild-type epitopes were manufactured at > 90% purity (Genscript, Piscataway, NJ). Reverse phase HPLC produced lyophilized peptides were reconstituted in DMSO (Sigma) and used to test T-cell reactivity.

### Monitoring of neoepitope-specific T-cell response

Mutated peptide specific T-cell response was investigated using T-cell presensitization method as previously described [84]. Briefly, CD8^+^ and CD4^+^ T-cells were sequentially isolated from PBMCs or tumors of EOC patients using Dynabeads CD8 and CD4 positive isolation kits (ThermoFisher Scientific) and incubated overnight in a 37 °C 5% CO_2_ incubator. Remaining CD4^−^CD8^−^ cells from PBMCs were used as APCs for pulsing a pool of 2 μM patient-specific mutated peptides overnight, irradiated at 3000 rad, washed and mixed with CD4^+^ or CD8^+^ T-cells. The CD4^+^ and CD8^+^ T-cells were cultured in the presence of 10 U/ml IL-2 (Roche) and 10 ng/ml IL-7 (R&D systems). A part of CD4^+^ T-cells were polyclonally activated by phytohemagglutinin (PHA, Remel-ThermoFisher Scientific) and cultured in the presence of IL-2 and IL-7 to prepare T-APC (antigen-presenting T-cells) [85]. At 13–15 days after the culture, these CD4^+^ and CD8^+^ T-cells were harvested and tested reactivity against individual mutated or wild-type peptides, or pooled peptides-loaded on autologous T-APC by IFN-γ ELISPOT assay as previously described [84]. Neoepitope-specific response was considered as positive with a minimum of 25 IFN-γ-spot-forming cells against mutated peptide per 5 × 10^4^ cells as well as the number of spots was 2-fold higher than the corresponding wild-type peptide [9].

### Establishment of neoepitope-specific T-cell clones

In order to characterize in details neoantigen recognition by neoepitope-specific T-cells, we established neoepitope-specific T-cell clones. For CD8^+^ T-cells, presensitized T-cells that showed neoantigen-specific reactivity in ELISPOT assays were restimulated by mutated peptide-pulsed T-APC and IFN-γ-producing T-cells were labeled using IFN-γ-capture reagent (Miltenyi Biotec) and sorted by flow-cytometry as described [86]. For CD4^+^ T-cells, presensitized T-cells were similarly restimulated in the presence of monensin (Sigma) and phycoerythrin (PE)-labeled anti-CD154 monoclonal antibody (mAb) as described [87] and CD154-expressing cells were sorted. Isolated T-cells were polyclonally expanded by PHA stimulation in the presence of allogeneic irradiated PBMC, IL-2 and IL-7. Purity and clonality of T-cells were tested by low-resolution TCR spectratyping using Vβ subtype-specific antibodies (Beckman Coulter). For some oligoclonal T-cell cultures containing different Vβ-expressing T-cells, cells were sorted again based on Vβ expression. Reactivity of neoepitope-specific T-cell clones were tested by ELISA and/or intracellular cytokine staining [88].

### Engineering neoepitope-specific T-cells by TCR gene transduction

Retroviral vectors expressing neoepitope-specific TCR genes were constructed as described previously with modifications [32]. Briefly, a part of sorted neoepitope-specific T-cells (2,000–5,000 T-cells) were lysed in TRIReagent. Total RNA was extracted by columns (Zymo Research), and reverse transcribed using SuperScript IV First-Strand Synthesis System (Thermo Fisher Scientific) using oligo dT primers according to the manufacturer’s instruction. Variable regions for TCR α and β chains were independently PCR-amplified using multiplexed primers, and assembled into a retroviral plasmid vector together with constant regions. Plasmids were amplified in NEBStable competent *E. coli* (New England Biolabs) and extracted using columns (ZymoResearch). To generate retroviral particles, GP2–293 packaging cell line (Clontech) was co-transfected with TCR-expressing transfer plasmid and envelope (pVSV-G: Clontech) plasmid using Lipofectamine 3000 reagent (Thermo Fisher Scientific). Medium was exchanged 7 h after transfection. Retroviral vectors were harvested at around 36 and 60 h after changing medium. PBMCs from healthy individuals were activated by PHA in the presence of IL-2, IL-7 and 10 ng/ml IL-12 (Peprotech). Activated T-cells were harvested at 36–48 h, and transduced by 125 μl freshly harvested retroviral vectors in a 96-well flat-bottom plate which was coated with Retronectin (TaKaRa Bio) and anti-CD3 mAb (OKT3; eBioscience). Transduction was repeated at 24 h after the first transduction on Retronectin without anti-CD3 mAb. In some experiments, we established PG13 clones stably producing high-titer retroviral vectors as described [89]. Briefly, PG13 (ATCC) was transduced with retroviral supernatant from GP2–293 and cloned by limiting dilution. To infect T-cells with PG13-derived retroviral particles, retroviral supernatant was added in a 96-well plate which was coated with Retronectin with or without anti-CD3 mAb and incubated for 6 h. After removing retroviral supernatant, the plate was rinsed by PBS containing 1% bovine serum albumin (BSA). Activated T-cells were transduced as described above. Function of transduced T-cells was investigated 3–7 days after the second transduction.

### Statistics

Statistical analysis was performed using R. Primary statistical tests include Student’s t test for single comparisons of normally distributed data, Chi-square test for comparsion of *BRCA1/2* mutation rate, Pearson’s correlation test for comparisons of APPM signature expression versus MYC expression, and Cox Proportional-Hazards Model to compare survival of TCGA patients with high versus low expression of APPM signature. All statistical tests are 2-tailed unless otherwise specified. A *p*-value less than 0.05 was considered significant.

## Additional files

Additional file 1: **Figure S1.** The protein view of selected genes with somatic mutations in the 20 EOC patients studied. **(a)** TP53. **(b)** BRCA1. **(c)** BRCA2. (d) IL27RA. Note that splicing site mutations (c.673-2A > G in TP53 and c.4987-1G > T in BRAC1) are not shown. (PPTX 130 kb)

Additional file 2: **Figure S2.** Comparison of somatic mutation and neoantigen between primary and local invasive tumors. **(a)** Somatic mutation. **(b)** Neoantigen. Pt #02 on the left and Pt #10 on the right. X-axis: variant allele frequency (VAF) in WES of primary tumor. Y-axis: VAF in WES of local invasive tumor. (PPTX 260 kb)

Additional file 3: **Figure S3.** Spontaneous CD4^+^ T-cell response against mutant-specific epitopes from autologous tumor-infiltrating lymphocytes (TIL) and/or peripheral blood lymphocytes (PBL). T-cell reactivity was measured by IFN-γ ELISpot assay (Methods). (−): no peptide; Wt: wildtype peptide; Mt.: Mutant peptide. (PPTX 85 kb)

Additional file 4: **Figure S4.** Spontaneous CD8^+^ T-cell response against mutant-specific epitopes from autologous tumor-infiltrating lymphocytes (TIL) and/or peripheral blood lymphocytes (PBL). T-cell reactivity was measured by IFN-γ ELISpot assay (Methods). (−): no peptide; Wt: wildtype peptide; Mt.: Mutant peptide. (PPTX 84 kb)

Additional file 5: **Figure S5.** The expression level of a panel of 10 immune inhibitory molecules from the tumor RNA-Seq data. These immune inhibitory molecules include PD1, PDL1, CTLA4, CD80, CD86, LAG3, TIM3, LAGLS9, MYC and FOXP3. The patients shown here include Pt #2, Pt #8, Pt #16 and Pt #5. (PPTX 78 kb)

Additional file 6: **Figure S6.** Recurrent somatic copy number alternations by the GISTC2.0 algorithm. GISTIC deletion (left) and amplification (right) plots using data from the five patients with mutant-specific T-cell response (top), and data from the five patients without mutant-specific T-cell response (bottom). The genome is oriented vertically from top to bottom, and GISTIC q-values at each locus are plotted from left to right on a log scale. The green line represents the default significance threshold (q-value = 0.25). For each plot, known or interesting cancer genes are highlighted. (PPTX 278 kb)

Additional file 7: **Figure S7.** TCR Vβ usage of NUP214 neopeptide-specific CD4^+^ T-cell line. **(a)** TCR Vβ usage of NUP214 neopeptide-specific CD4^+^ T-cell line was determined by flow cytometry. **(b)** NUP214 neopeptide-specific IFN-γ production from Vβ2^+^, Vβ13.1^+^, or Vβ2^−^Vβ13.1^−^ T-cells was examined by intracellular cytokine staining. (PPTX 221 kb)

Additional file 8: **Figure S8.** Characterization of JAK1 neoepitope-specific CD4^+^ T-cells. **(a)** Peptide reactivity of a *JAK1* neoepitope-specific CD4^+^ T-cell line. IFN-γ and GM-CSF production on CD4^+^ T-cells against JAK1 mutated (IEILRNLYHEIIV) or wild-type (IEILRNLYHENIV) peptide-pulsed autologous EBV-B-cells were determined by intracellular cytokine staining. **(b)** TCR usage of JAK1 neoepitope-specific CD4^+^ T-cell line. T-cells were stained with TCR Vβ subtype-specific antibodies and analyzed by flow cytometry. **(c)** Purity of Vβ13.6^+^ cells after magnetic-beads sorting. **(d)** Avidity of JAK1 neoepitope-specific T-cell clone. CD4^+^ T-cell clones were stimulated with autologous EBV-B-cells pulsed with the indicated concentration of mutated or wild-type peptide for 6 h in the presence of Golgi stop. IFN-γ production from Vβ13.6^+^ cells were determined by flow cytometry. The data represents mean ± s.d. of duplicate wells. **(e)** Recognition of autologous tumor-derived cells by Vβ13.6^+^ T-cell clone. PBMC or AMC were co-cultured with Vβ13.6^+^ JAK1 neoepitope-specific CD4^+^ T clones or without T-cells (−) for 24 h. AMC: ascites-derived mononuclear cells. IFN-γ production was measured by ELISA. The data represent mean + s.d. of duplicate wells. *p < 0.05 compared to IFN-γ level against PBMC. (PPTX 250 kb)

Additional file 9: **Figure S9.** Reactivity of NUP214 neoepitope-specific TCR-transduced CD8^+^ and CD4^+^ T-cells. IFN-γ production **(a)** and GM-CSF **(b)** production from Vβ13.1^+^ or Vβ2^−^Vβ13.1^−^ TCR- transduced CD8^+^ and CD8^−^ (CD4^+^) T-cells against EBV-B-cells pulsed with or without NUP214 mutated or wild-type peptide were determined by intracellular cytokine staining. (PPTX 683 kb)

Additional file 10: **Figure S10.** Analysis of TRPC4 neoepitope-specific CD8^+^ T-cell clone. **(a)** IFN-γ and GM-CSF production on CD8^+^ T-cells against TRPC4 mutated (QSLFWSIFV) or wild-type (QSLFWSIFG) peptide-pulsed autologous EBV-B-cells were determined by intracellular cytokine staining. **(b)** Avidity of TRPC4 neoepitope-specific T-cell clone. CD8^+^ T-cell clone was co-cultured with autologous EBV-B-cells pulsed with the different concentration of mutated or wild-type peptide for 24 h. IFN-γ level in the culture supernatant was measured by ELISA. The data represents mean ± s.d. of duplicate wells. **(c)** IFN-γ production from TRPC4 neoepitope-specific TCR-transduced T-cells against EBV-B pulsed with or without TRPC4 mutated or wild-type peptide was determined by intracellular cytokine staining. (PPTX 321 kb)

Additional file 11: **Figure S11.** The expression level of APPM signature from the tumor RNA-Seq data. The APPM expression is derived from the median expression value (in RPKM) of the 31 genes within the APPM signature (Methods). The patients shown here include Pt #3, Pt #15, Pt #19 and Pt #5. (PPTX 50 kb)

Additional file 12: **Table S1.** Clinical demographics of treatment-naïve (including both chemotherapy and immunotherapy) epithelial ovarian cancer patients at the time of primary debulking surgery. Patient identification number, age at diagnosis, tumor histologic type, FIGO stage, debulking status, residual tumor mass after debulking surgery, number of recurrences after primary debulking surgery, and RECIST to frontline chemotherapy after primary debulking surgery. (XLSX 10 kb)

Additional file 13: **Table S2.** Somatic point mutations identified from whole-exome sequencing. **(a)** The 18 patients with either primary tumor or locally invasive tumor; **(b)** The 2 patients with both primary tumor and locally invasive tumor. AA, amino acid; CGC, cancer gene census; VAF, variant allele frequency. (XLSX 244 kb)

Additional file 14: **Table S3.** Somatic mutation burdens and predicted neoantigen load in the 20 patients. The predicted neoantigens are classified as expressed or non-expressed based on the mutant allele’s expression level in RNAseq data (see Method section). (XLSX 10 kb)

Additional file 15: **Table S4.** Description of the 75 neopeptides screened for immunogenicity. The expression status is based on the mutant allele’s expression level in RNAseq data, and affinity score is predicted by NetMHC algorithm with default setting (see Method section). (XLSX 15 kb)

Additional file 16: **Table S5.** The list of 31 genes within the derived APPM signature (see Method section). (XLSX 11 kb)

## Data Availability

The datasets generated and/or analyzed during the current study are available from the corresponding author on reasonable request.

## Abbreviations

AA: Amino acid
ACT: Adoptive cell therapy
AMC: Ascites mononuclear cells
APC: Antigen presenting cells
APPM: Antigen processing and presentation machinery
BSA: Bovine serum albumin
CGC: Cancer gene census
CLP: Christmas light plot
DEG: Differentially expressed genes
DMSO: Dimethyl sulfoxide
EBV: Epstein-Barr virus
ELISpot: Enzyme-linked immune absorbent spot
EOC: Epithelial ovarian cancer
FIGO: International Federation of Gynaecology and Obstetrics
GM-CSF: Granulocyte macrophage colony stimulating factor
HLA: Human leukocyte antigen
HPLC: High-performance liquid chromatography
IFN: Interferon
IL: Interleukin
mAb: Monoclonal antibody
MHC: Major histocompatibility complex
PBL: Peripheral blood lymphocytes
PBMC: Peripheral blood mononuclear cells
PBS: Phosphate buffered saline
Pt#: Patient number
RECIST: Response Evaluation Criteria In Solid Tumors
RNAseq: RNA sequencing
s.d.: standard deviation
SNV: Single nucleotide variants
TCGA: The Cancer Genome Atlas
TCR: T-cell receptor
TIL: Tumor infiltrating lymphocytes
TMC: Tumor mononuclear cells
VAF: Variant allele frequency
WES: Whole exome sequencing
