# Supplementary figures and images for: Efficient identification of neoantigen-specific T-cell responses in advanced human ovarian cancer
Source: J Immunother Cancer. 2019 Jun 20;7:156. doi: 10.1186/s40425-019-0629-6 (PMC6587259; doi:10.1186/s40425-019-0629-6)

## Slide 1
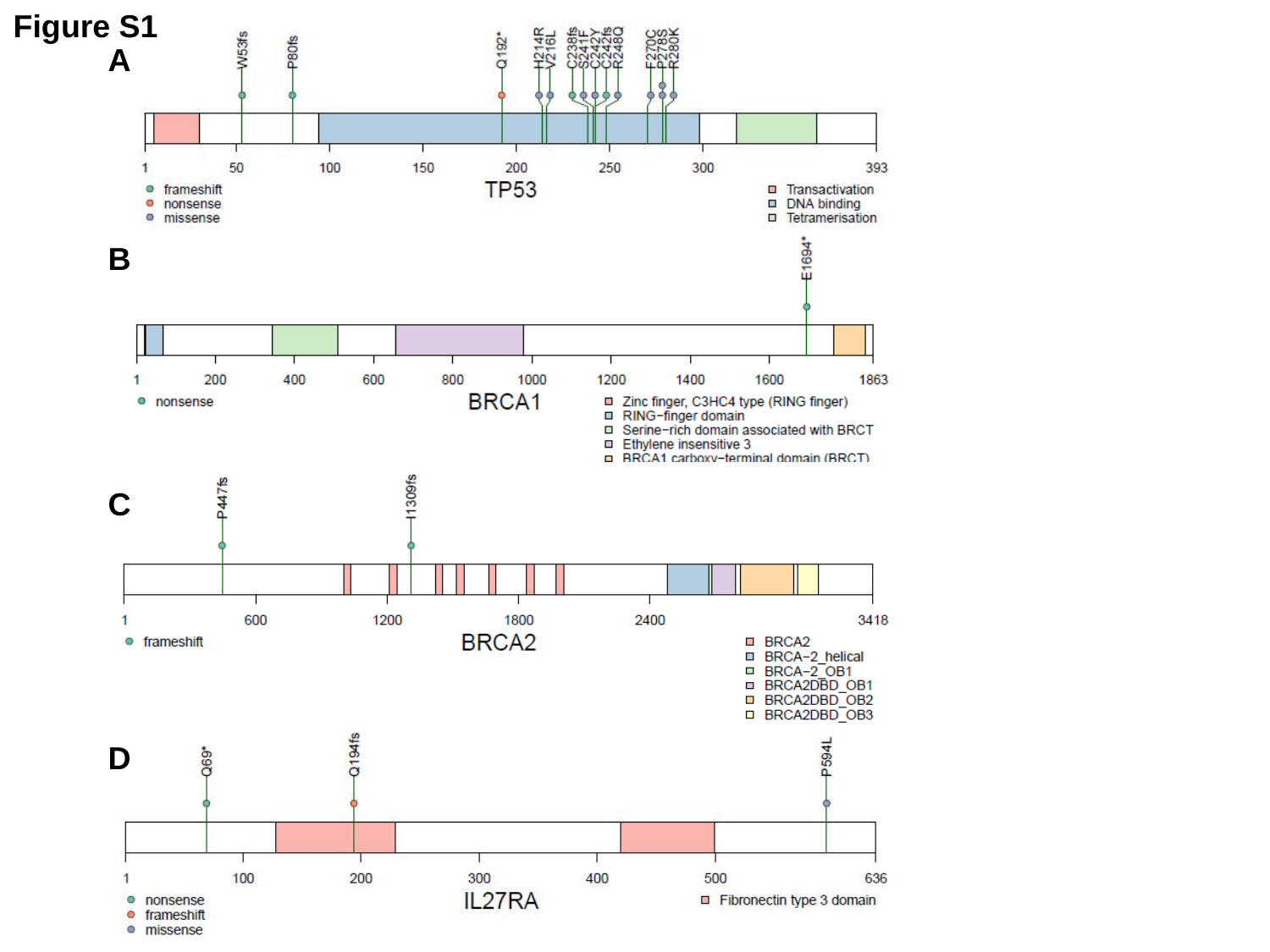

Figure S1
A
B
C
D

Supplement: Supplementary file 1 — Figure S1. The protein view of selected genes with somatic mutations in the 20 EOC patients studied. (a) TP53. (b) BRCA1. (c) BRCA2. (d) IL27RA. Note that splicing site mutations (c.673-2A > G in TP53 and c.4987-1G > T in BRAC1) are not shown. (PPTX 130 kb) [file 40425_2019_629_MOESM1_ESM.pptx]

## Slide 1
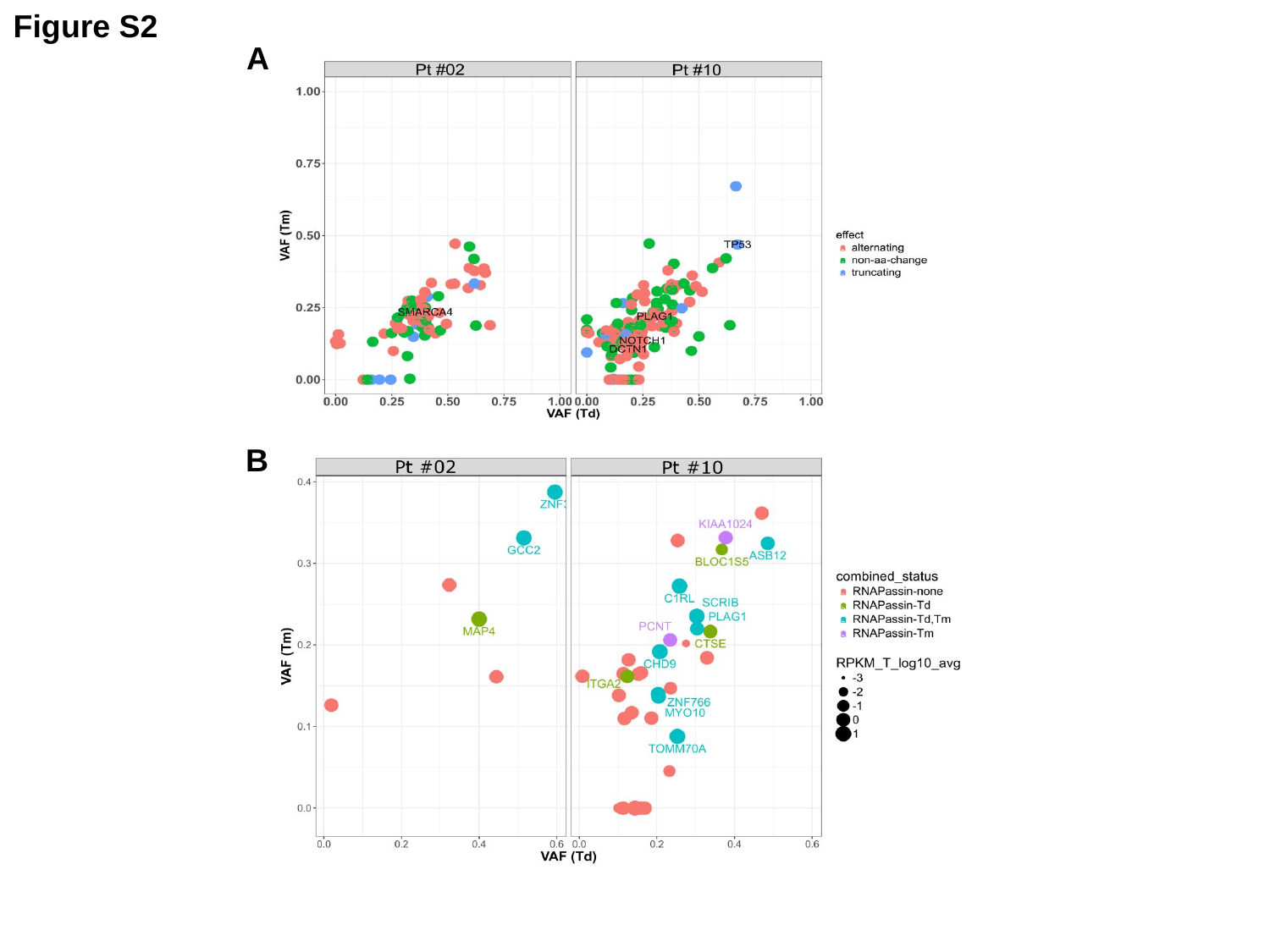

Figure S2
A
B

Supplement: Supplementary file 2 — Figure S2. Comparison of somatic mutation and neoantigen between primary and local invasive tumors. (a) Somatic mutation. (b) Neoantigen. Pt #02 on the left and Pt #10 on the right. X-axis: variant allele frequency (VAF) in WES of primary tumor. Y-axis: VAF in WES of local invasive tumor. (PPTX 260 kb) [file 40425_2019_629_MOESM2_ESM.pptx]

## Slide 1
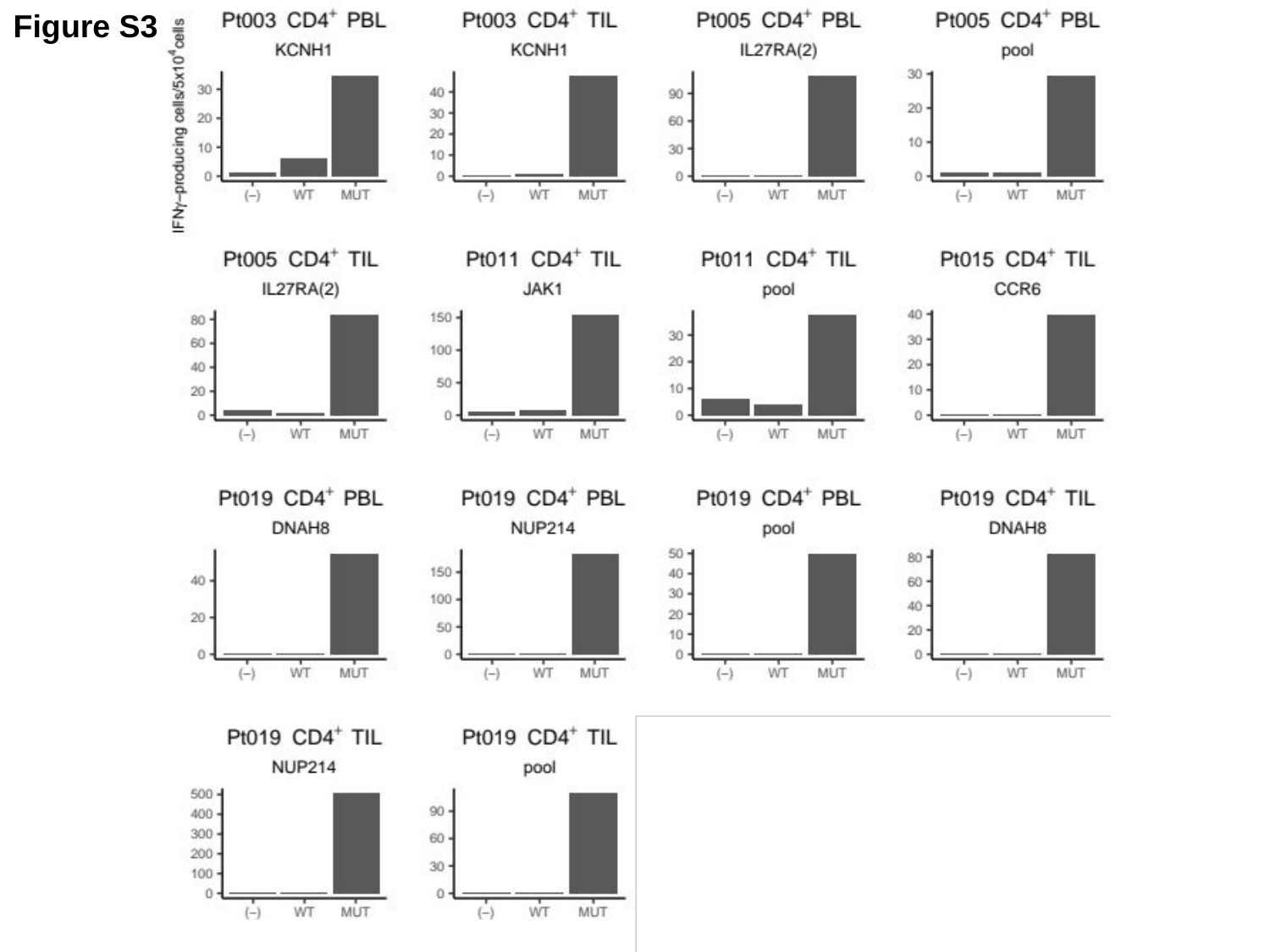

Figure S3

Supplement: Supplementary file 3 — Figure S3. Spontaneous CD4+ T-cell response against mutant-specific epitopes from autologous tumor-infiltrating lymphocytes (TIL) and/or peripheral blood lymphocytes (PBL). T-cell reactivity was measured by IFN-γ ELISpot assay (Methods). (−): no peptide; Wt: wildtype peptide; Mt.: Mutant peptide. (PPTX 85 kb) [file 40425_2019_629_MOESM3_ESM.pptx]

## Slide 1
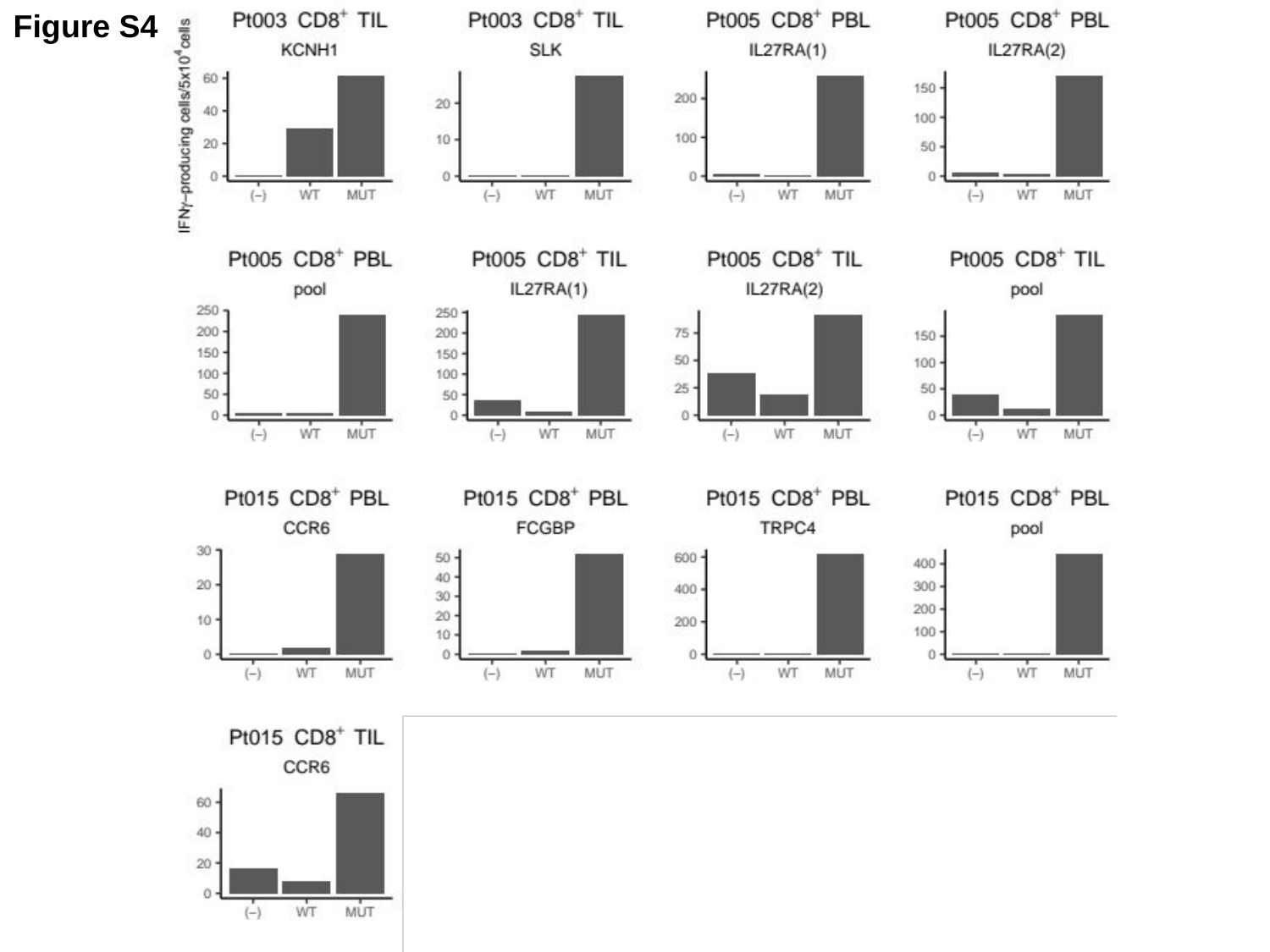

Figure S4

Supplement: Supplementary file 4 — Figure S4. Spontaneous CD8+ T-cell response against mutant-specific epitopes from autologous tumor-infiltrating lymphocytes (TIL) and/or peripheral blood lymphocytes (PBL). T-cell reactivity was measured by IFN-γ ELISpot assay (Methods). (−): no peptide; Wt: wildtype peptide; Mt.: Mutant peptide. (PPTX 84 kb) [file 40425_2019_629_MOESM4_ESM.pptx]

## Slide 1
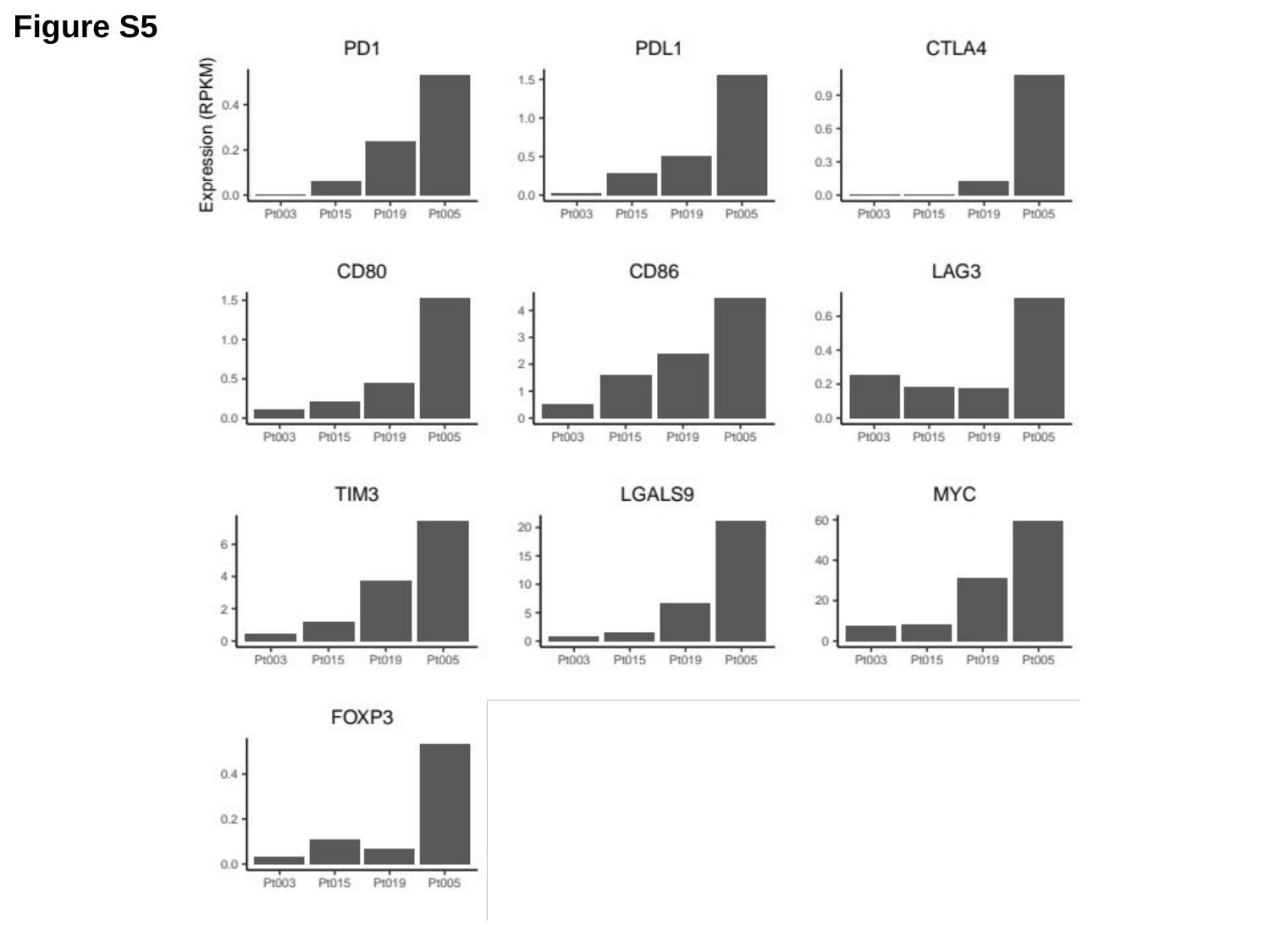

Figure S5

Supplement: Supplementary file 5 — Figure S5. The expression level of a panel of 10 immune inhibitory molecules from the tumor RNA-Seq data. These immune inhibitory molecules include PD1, PDL1, CTLA4, CD80, CD86, LAG3, TIM3, LAGLS9, MYC and FOXP3. The patients shown here include Pt #2, Pt #8, Pt #16 and Pt #5. (PPTX 78 kb) [file 40425_2019_629_MOESM5_ESM.pptx]

## Slide 1
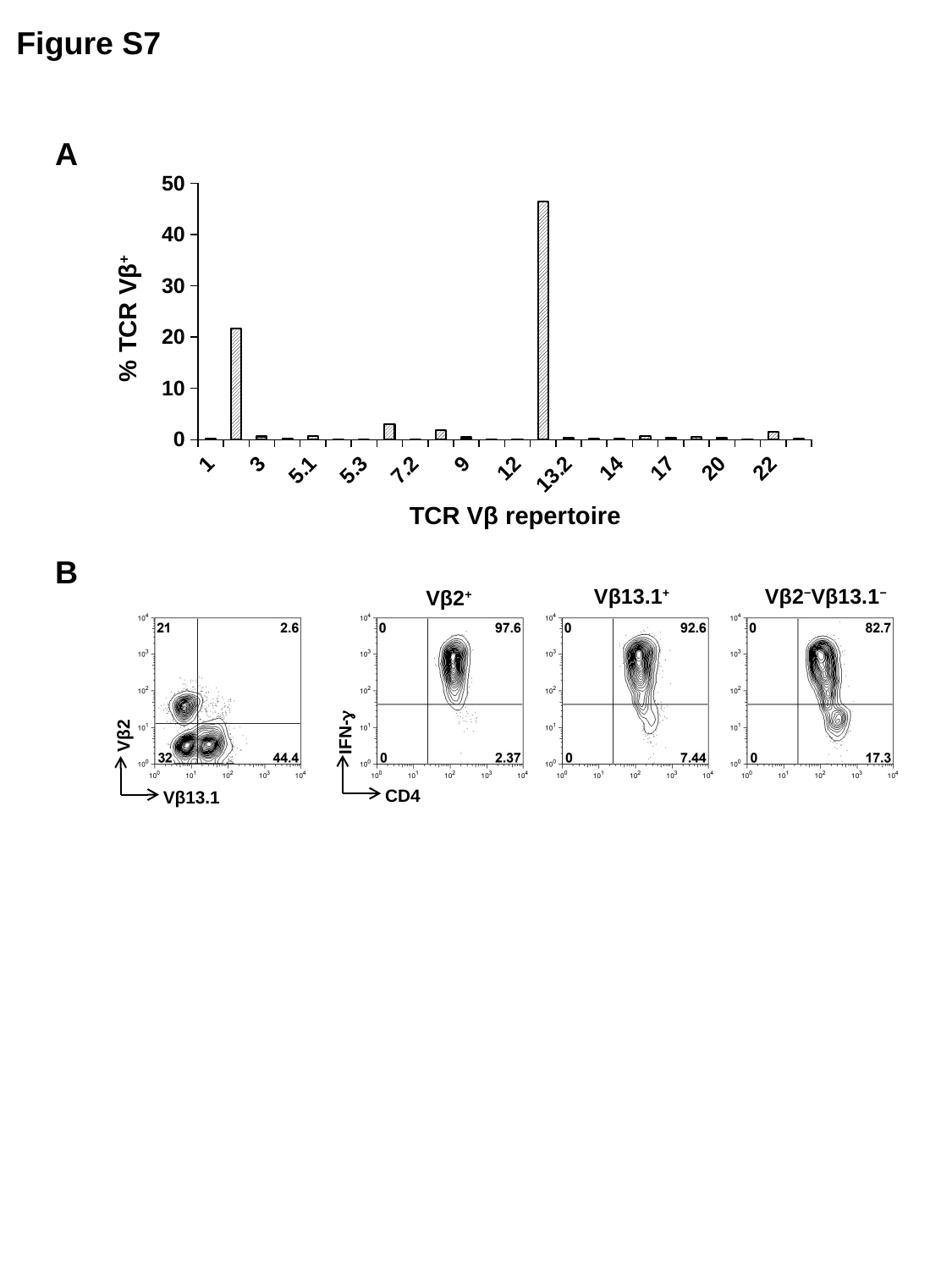

Figure S7
A
### Chart
| Category | CD4-T |
|---|---|
| 1 | 0.28 |
| 2 | 21.7 |
| 3 | 0.62 |
| 4 | 0.13 |
| 5.0999999999999996 | 0.74 |
| 5.2 | 0.065 |
| 5.3 | 0.054 |
| 7.1 | 2.96 |
| 7.2 | 0.021 |
| 8 | 1.85 |
| 9 | 0.45 |
| 11 | 0.096 |
| 12 | 0.11 |
| 13.1 | 46.4 |
| 13.2 | 0.37 |
| 13.6 | 0.14 |
| 14 | 0.18 |
| 16 | 0.68 |
| 17 | 0.32 |
| 18 | 0.58 |
| 20 | 0.32 |
| 21.3 | 0.11 |
| 22 | 1.57 |
| 23 | 0.18 |% TCR Vβ+
TCR Vβ repertoire
B
Vβ13.1+
Vβ2−Vβ13.1−
Vβ2+
IFN-γ
Vβ2
CD4
Vβ13.1

Supplement: Supplementary file 7 — Figure S7. TCR Vβ usage of NUP214 neopeptide-specific CD4+ T-cell line. (a) TCR Vβ usage of NUP214 neopeptide-specific CD4+ T-cell line was determined by flow cytometry. (b) NUP214 neopeptide-specific IFN-γ production from Vβ2+, Vβ13.1+, or Vβ2−Vβ13.1− T-cells was examined by intracellular cytokine staining. (PPTX 221 kb) [file 40425_2019_629_MOESM7_ESM.pptx]

## Slide 1
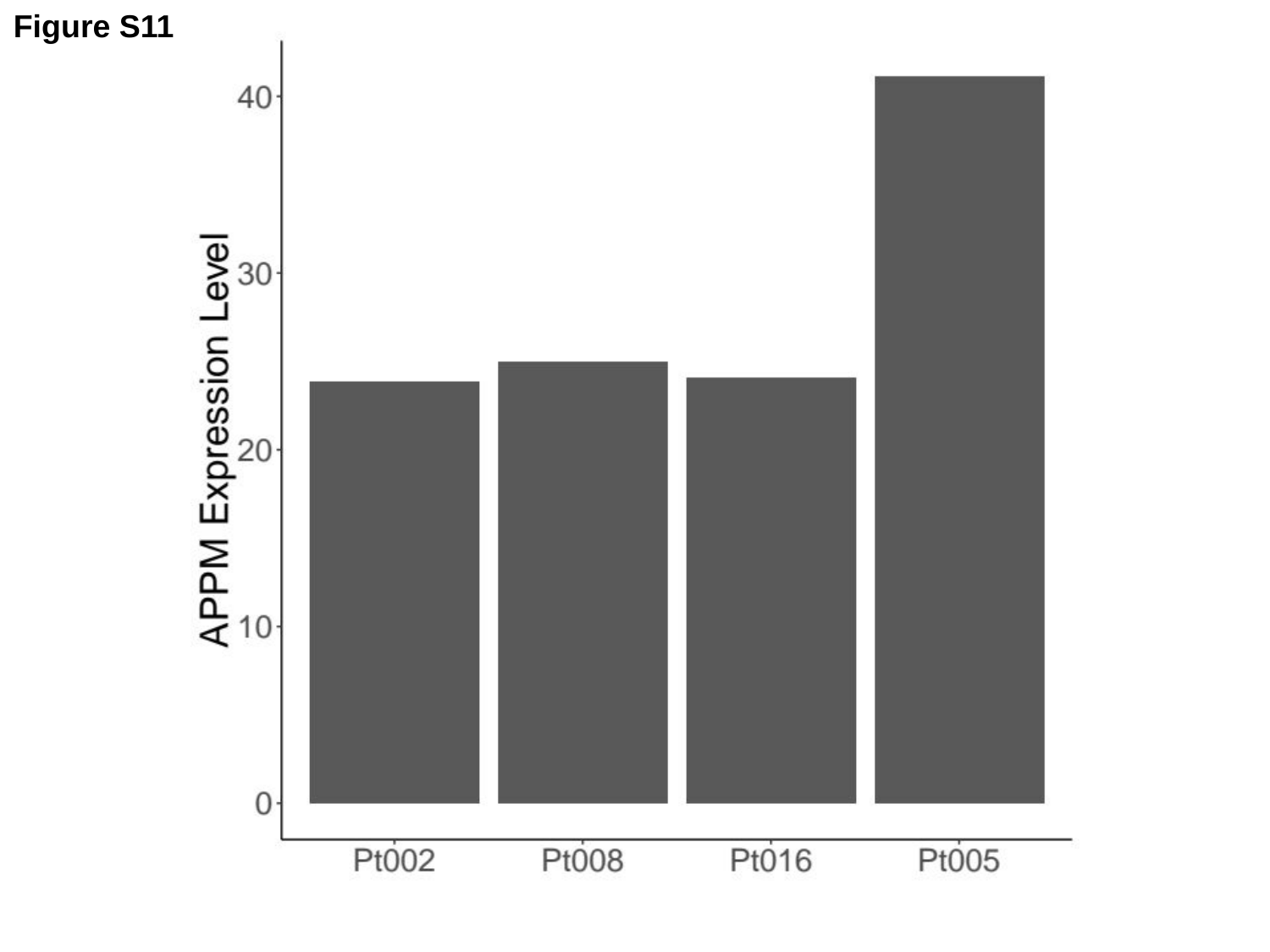

Figure S11

Supplement: Supplementary file 11 — Figure S11. The expression level of APPM signature from the tumor RNA-Seq data. The APPM expression is derived from the median expression value (in RPKM) of the 31 genes within the APPM signature (Methods). The patients shown here include Pt #3, Pt #15, Pt #19 and Pt #5. (PPTX 50 kb) [file 40425_2019_629_MOESM11_ESM.pptx]
