# Supplementary material for: Efficient identification of neoantigen-specific T-cell responses in advanced human ovarian cancer
Source: J Immunother Cancer. 2019 Jun 20;7:156. doi: 10.1186/s40425-019-0629-6 (PMC6587259; doi:10.1186/s40425-019-0629-6)
Supplement: Supplementary file 6 — Figure S6. Recurrent somatic copy number alternations by the GISTC2.0 algorithm. GISTIC deletion (left) and amplification (right) plots using data from the five patients with mutant-specific T-cell response (top), and data from the five patients without mutant-specific T-cell response (bottom). The genome is oriented vertically from top to bottom, and GISTIC q-values at each locus are plotted from left to right on a log scale. The green line represents the default significance threshold (q-value = 0.25). For each plot, known or interesting cancer genes are highlighted. (PPTX 278 kb) [file 40425_2019_629_MOESM6_ESM.pptx]

## Slide 1
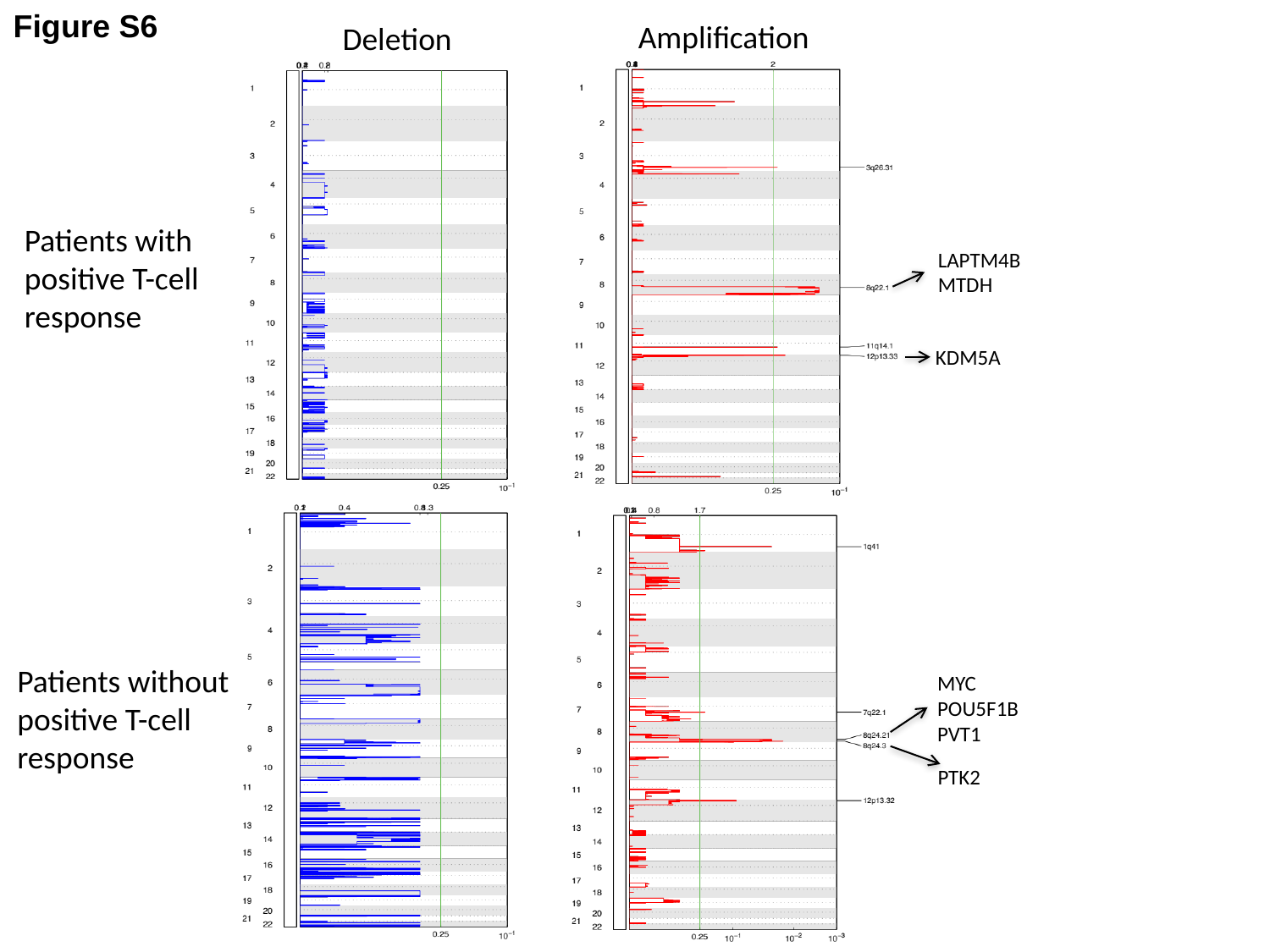

Figure S6
Amplification
Deletion
Patients with
positive T-cell
response
LAPTM4B
MTDH
KDM5A
Patients without
positive T-cell
response
MYC
POU5F1B
PVT1
PTK2
