# Supplementary material for: Efficient identification of neoantigen-specific T-cell responses in advanced human ovarian cancer
Source: J Immunother Cancer. 2019 Jun 20;7:156. doi: 10.1186/s40425-019-0629-6 (PMC6587259; doi:10.1186/s40425-019-0629-6)
Supplement: Supplementary file 8 — Figure S8. Characterization of JAK1 neoepitope-specific CD4+ T-cells. (a) Peptide reactivity of a JAK1 neoepitope-specific CD4+ T-cell line. IFN-γ and GM-CSF production on CD4+ T-cells against JAK1 mutated (IEILRNLYHEIIV) or wild-type (IEILRNLYHENIV) peptide-pulsed autologous EBV-B-cells were determined by intracellular cytokine staining. (b) TCR usage of JAK1 neoepitope-specific CD4+ T-cell line. T-cells were stained with TCR Vβ subtype-specific antibodies and analyzed by flow cytometry. (c) Purity of Vβ13.6+ cells after magnetic-beads sorting. (d) Avidity of JAK1 neoepitope-specific T-cell clone. CD4+ T-cell clones were stimulated with autologous EBV-B-cells pulsed with the indicated concentration of mutated or wild-type peptide for 6 h in the presence of Golgi stop. IFN-γ production from Vβ13.6+ cells were determined by flow cytometry. The data represents mean ± s.d. of duplicate wells. (e) Recognition of autologous tumor-derived cells by Vβ13.6+ T-cell clone. PBMC or AMC were co-cultured with Vβ13.6+ JAK1 neoepitope-specific CD4+ T clones or without T-cells (−) for 24 h. AMC: ascites-derived mononuclear cells. IFN-γ production was measured by ELISA. The data represent mean + s.d. of duplicate wells. *p < 0.05 compared to IFN-γ level against PBMC. (PPTX 250 kb) [file 40425_2019_629_MOESM8_ESM.pptx]

## Slide 1
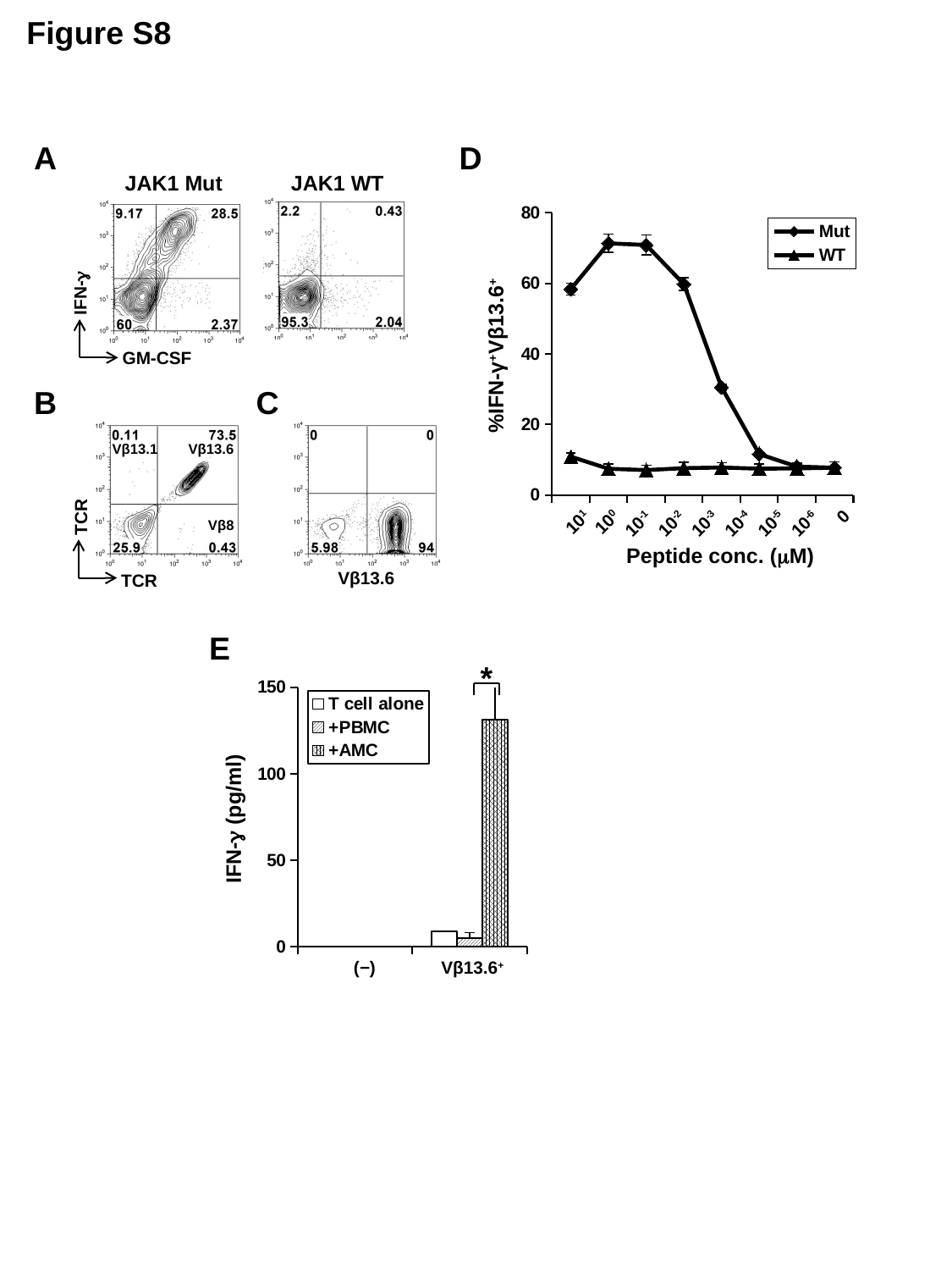

Figure S8
A
D
JAK1 Mut
JAK1 WT
### Chart
| Category | Mut | WT |
|---|---|---|
| 10 | 58.33333333333334 | 10.84333333333333 |
| 1 | 71.3 | 7.43 |
| 0.1 | 70.8333333333333 | 7.053333333333334 |
| 0.01 | 59.73333333333333 | 7.596666666666667 |
| 1E-3 | 30.53333333333331 | 7.780000000000001 |
| 1E-4 | 11.6 | 7.44 |
| 1.0000000000000001E-5 | 8.033333333333333 | 7.526666666666666 |
| 0 | 7.733333333333336 | 7.733333333333336 |
IFN-γ
%IFN-γ+Vβ13.6+
GM-CSF
B
C
Vβ13.1
Vβ13.6
0
TCR
101
100
10-1
10-2
10-3
10-4
10-5
10-6
Vβ8
Peptide conc. (μM)
Vβ13.6
TCR
E
*
### Chart
| Category | T cell alone | +PBMC | +AMC |
|---|---|---|---|
| (-) | None | 0.0 | 0.0 |
| Vb13.6+ | 8.841200469999999 | 4.969131658315949 | 131.1730821743531 |IFN-γ (pg/ml)
(−)
Vβ13.6+
