# Supplementary material for: Efficient identification of neoantigen-specific T-cell responses in advanced human ovarian cancer
Source: J Immunother Cancer. 2019 Jun 20;7:156. doi: 10.1186/s40425-019-0629-6 (PMC6587259; doi:10.1186/s40425-019-0629-6)
Supplement: Supplementary file 9 — Figure S9. Reactivity of NUP214 neoepitope-specific TCR-transduced CD8+ and CD4+ T-cells. IFN-γ production (a) and GM-CSF (b) production from Vβ13.1+ or Vβ2−Vβ13.1− TCR- transduced CD8+ and CD8− (CD4+) T-cells against EBV-B-cells pulsed with or without NUP214 mutated or wild-type peptide were determined by intracellular cytokine staining. (PPTX 683 kb) [file 40425_2019_629_MOESM9_ESM.pptx]

## Slide 1
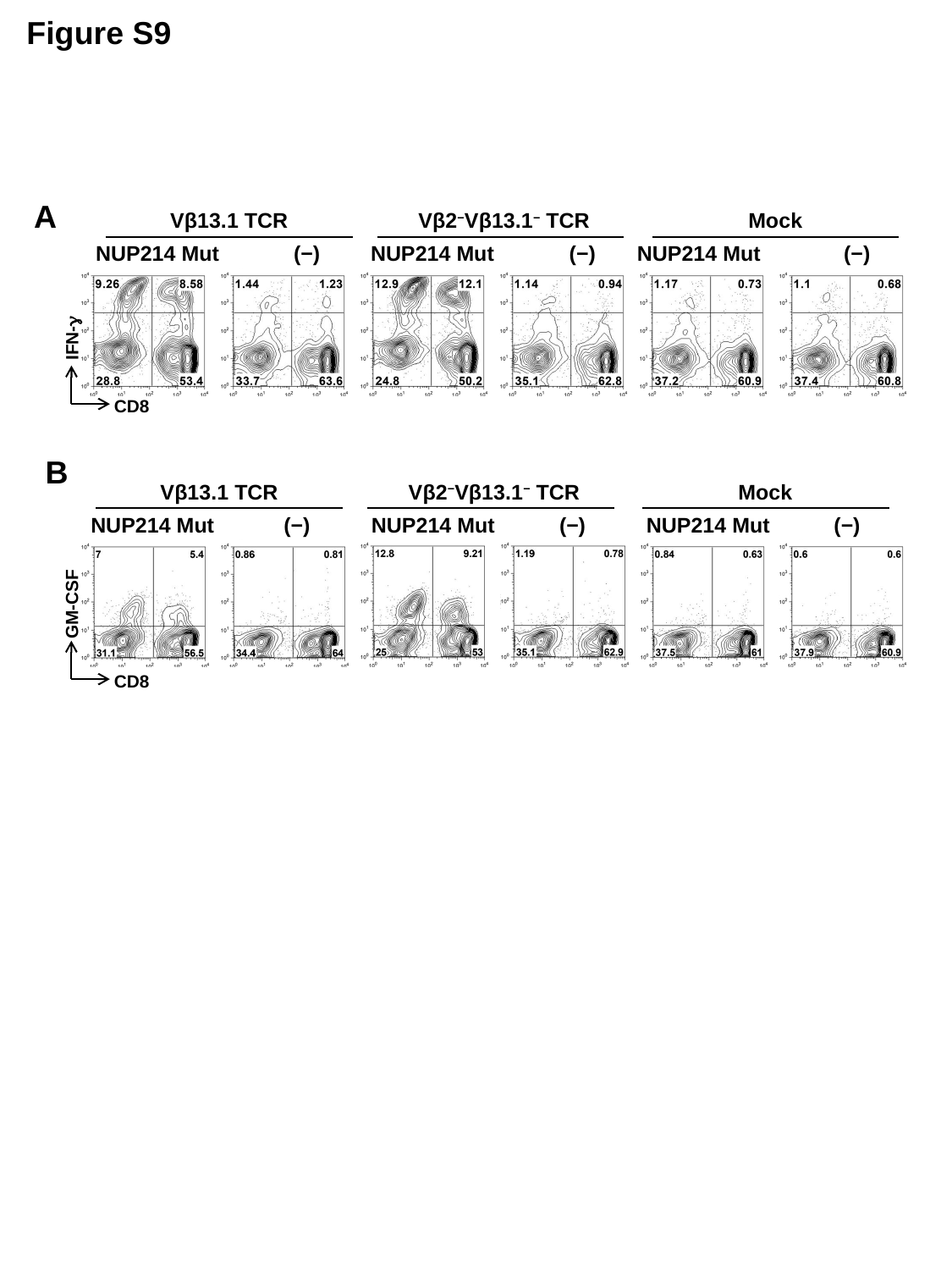

Figure S9
A
Vβ13.1 TCR
Vβ2−Vβ13.1− TCR
Mock
NUP214 Mut
(−)
NUP214 Mut
(−)
NUP214 Mut
(−)
IFN-γ
CD8
B
Vβ13.1 TCR
Vβ2−Vβ13.1− TCR
Mock
NUP214 Mut
(−)
NUP214 Mut
(−)
NUP214 Mut
(−)
GM-CSF
CD8
