# Supplementary material for: Efficient identification of neoantigen-specific T-cell responses in advanced human ovarian cancer
Source: J Immunother Cancer. 2019 Jun 20;7:156. doi: 10.1186/s40425-019-0629-6 (PMC6587259; doi:10.1186/s40425-019-0629-6)
Supplement: Supplementary file 10 — Figure S10. Analysis of TRPC4 neoepitope-specific CD8+ T-cell clone. (a) IFN-γ and GM-CSF production on CD8+ T-cells against TRPC4 mutated (QSLFWSIFV) or wild-type (QSLFWSIFG) peptide-pulsed autologous EBV-B-cells were determined by intracellular cytokine staining. (b) Avidity of TRPC4 neoepitope-specific T-cell clone. CD8+ T-cell clone was co-cultured with autologous EBV-B-cells pulsed with the different concentration of mutated or wild-type peptide for 24 h. IFN-γ level in the culture supernatant was measured by ELISA. The data represents mean ± s.d. of duplicate wells. (c) IFN-γ production from TRPC4 neoepitope-specific TCR-transduced T-cells against EBV-B pulsed with or without TRPC4 mutated or wild-type peptide was determined by intracellular cytokine staining. (PPTX 321 kb) [file 40425_2019_629_MOESM10_ESM.pptx]

## Slide 1
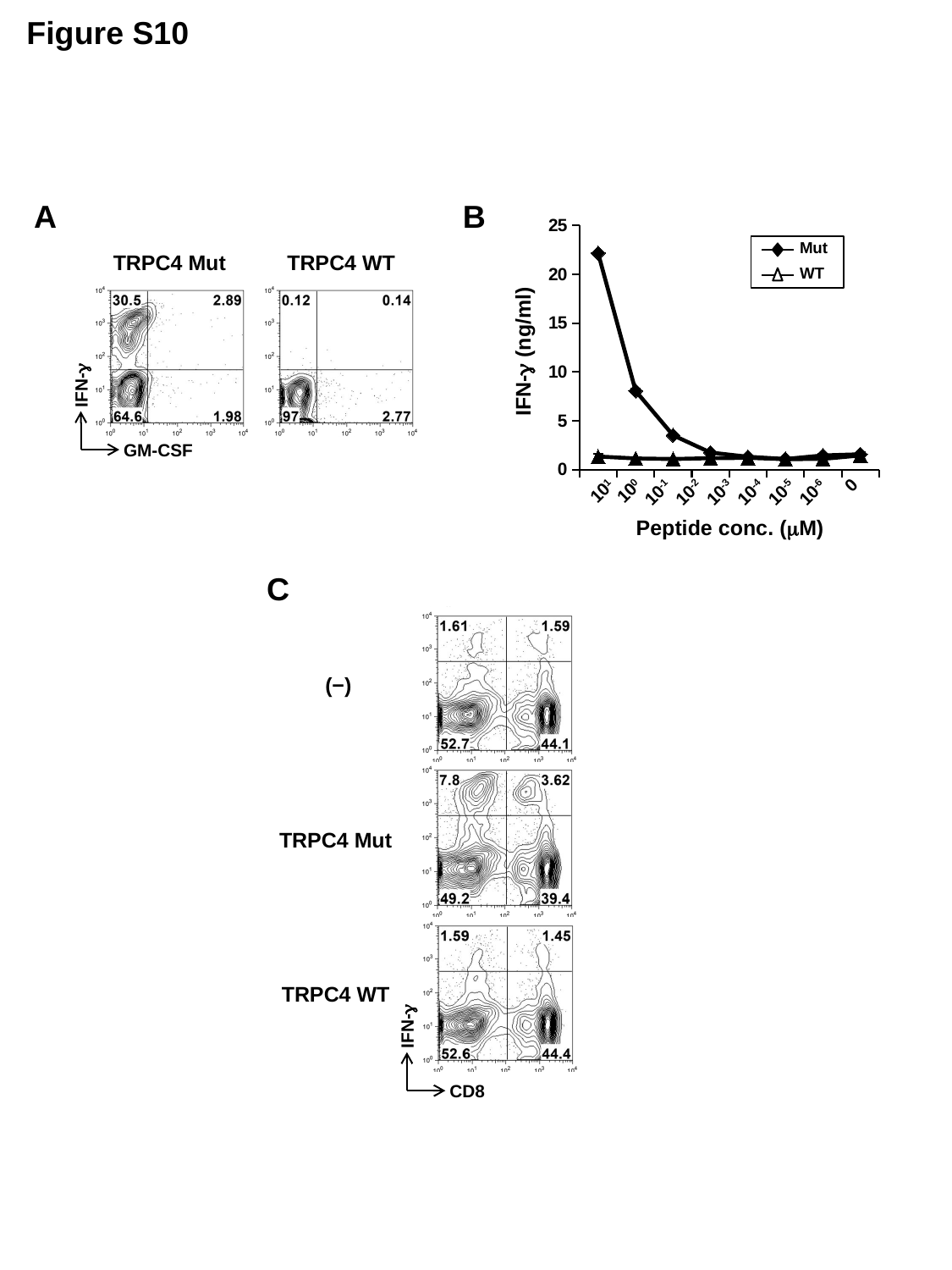

Figure S10
A
B
### Chart
| Category | 103 | 104 |
|---|---|---|
| 10^1 | 22.15984366646236 | 1.34378832123605 |
| 10^0 | 8.05765899151295 | 1.141882845324507 |
| 10^-1 | 3.513819878812471 | 1.089593937937259 |
| 10^-2 | 1.74968751789539 | 1.168574514162746 |
| 10^-3 | 1.317110985199855 | 1.203081040781138 |
| 10^-4 | 1.089572753665953 | 1.072250473352994 |
| 10^-5 | 1.449055393393999 | 1.098463907228466 |
| 10^-6 | 1.572595060909377 | 1.449382363491514 |Mut
WT
TRPC4 Mut
TRPC4 WT
IFN-γ (ng/ml)
IFN-γ
GM-CSF
0
101
100
10-1
10-2
10-3
10-4
10-5
10-6
Peptide conc. (μM)
C
(−)
TRPC4 Mut
TRPC4 WT
IFN-γ
CD8
